# Personalized ctDNA monitoring in metastatic HR+/HER2− breast cancer patients during endocrine and CDK4/6 inhibitor therapy

**DOI:** 10.1038/s41523-025-00783-2

**Published:** 2025-07-19

**Authors:** Jesús Fuentes-Antrás, Mitchell J. Elliott, Sasha C. Main, Philippe Echelard, Aaron Dou, Philippe L. Bedard, Eitan Amir, Michelle B. Nadler, Nicholas Meti, Nancy Gregorio, Elizabeth Shah, Emily Van de Laar, Celeste Yu, Yangqing Deng, Lisa Gates, Clodagh Murray, Christopher G. Smith, Amber Chevalier, Scott V. Bratman, Lillian L. Siu, Hal K. Berman, David W. Cescon

**Affiliations:** 1 Division of Medical Oncology & Hematology, Department of Medicine, Princess Margaret Cancer Centre and University of Toronto, Toronto, ON Canada; 2 NEXT Oncology, Experimental Therapeutics Unit, Hospital Universitario Quirónsalud Madrid, Madrid, Spain; 3 Department of Medical Biophysics, University of Toronto, Toronto, ON Canada; 4 Princess Margaret Cancer Centre, University Health Network, Toronto, ON Canada; 5 Department of Pathology and Laboratory Medicine, University Health Network, Toronto, ON Canada; 6 Gerald Bronfman Department of Oncology, St. Mary’s Hospital Center, McGill University, Montreal, QC Canada; 7 Department of Statistics, University of Toronto, Toronto, ON Canada; 8 NeoGenomics, Inc, Fort Myers, FL USA; 9 Department of Radiation Oncology, University of Toronto, Toronto, ON Canada

**Keywords:** Breast cancer, Biomarkers

## Abstract

Improved methods to monitor treatment response may enhance patient management and clinical outcomes. This study assessed the feasibility and performance of a tumor-informed circulating tumor DNA (ctDNA) assay in metastatic HR+/HER2− breast cancer patients receiving endocrine and CDK4/6 inhibitor therapy. By conducting whole exome sequencing on archival tumors, highly sensitive personalized ctDNA panels were designed for blood monitoring. The assay showed high detection sensitivity (91% baseline, 70% all timepoints) and associations between higher baseline estimated variant allele fractions, liver metastases, and shorter time to treatment failure (TTF) and overall survival (OS). Complete molecular response, defined as ctDNA clearance, was observed in 28% of patients and correlated with improved TTF (HR 0.07) and OS (HR 0.07). The last cleared timepoint predated treatment failure by a median 14.3 months. ctDNA rises or limited decreases preceded radiographic progression. Molecular metrics may facilitate plasma-first monitoring and innovative strategies for clinical practice and trial design.

## Introduction

Circulating tumor DNA (ctDNA) has garnered increasing attention as a biomarker capable of delivering prognostic and predictive insights, thereby informing therapeutic decisions and surveillance strategies in oncology^1,2^. As a component of cell-free DNA (cfDNA) found within the bloodstream, ctDNA is genetic material shed from tumor cells and presents a minimally invasive alternative to traditional tissue biopsies for molecular characterization. ctDNA levels are also associated with disease burden and treatment response, and thus hold promise for advancing the paradigm of precision oncology by enabling disease and treatment response monitoring. Serial ctDNA quantification could permit personalized follow-up and allow tailoring of therapeutic strategies to a patient’s unique clonal architecture and ctDNA kinetics.

Despite its potential advantages, the association between ctDNA changes and clinical outcomes in metastatic breast cancer (MBC) remains uncertain. This limitation may be primarily attributed to technical variability and a lack of comprehensive studies with serially collected samples analyzed with fit-for-purpose approaches. In particular, limited longitudinal ctDNA data are available for HR+/HER2− MBC patients receiving CDK4/6 inhibitors (CDK4/6i) with endocrine therapy (ET), where the median progression-free survival is ~2–3 years. Most reports have focused on early ctDNA changes to establish associations with clinical outcomes, rather than attempting to capture ctDNA dynamics throughout the entire treatment course^3–6^. Many studies have not performed concurrent tumor measurements utilizing the Response Evaluation Criteria in Solid Tumors version 1.1 (RECIST v1.1), which impedes the evaluation of the correlation of ctDNA quantification and tumor burden changes. Furthermore, these studies employed a range of tumor-agnostic, single-nucleotide variant (SNV)-based ctDNA methodologies, including next-generation sequencing (NGS) panels and polymerase chain reaction (PCR) of gene alterations of interest (e.g., *PIK3CA*, *ESR1*) with varying sensitivities that may depend on tumor genotypes^7^. A substantial rate of false negatives —with ctDNA levels that may be below the limit of detection— may render existing tumor-agnostic assays insufficient for accurately measuring tumor kinetics. This phenomenon adversely impacts risk stratification and, importantly, robust ctDNA tracking, hindering the ability to capture on-treatment ctDNA responses and the identification of biomarkers with clinically meaningful lead times to clinical outcomes of interest. This challenge is further exacerbated by the relatively low ctDNA shedding rate in breast cancer, particularly in the hormone receptor-positive subtype, and by the presence of variants arising from clonal hematopoiesis of indeterminate potential (CHIP) which may be misinterpreted as tumor-derived ctDNA^8^.

To address some of these shortcomings, tumor-informed ctDNA analysis, which leverages mutational information from the primary tumor to enhance ctDNA detection, holds promise in improving sensitivity, specificity, and prognostic/predictive capabilities. This approach is predicated on the premise that truncal tumor mutations—whether drivers or passengers—are common to all cancer cells, regardless of subclonal mutations mediating resistance, and in aggregate increase sensitivity of detection in plasma^9^. Tumor-informed ctDNA assays have demonstrated potential to improve minimal residual disease (MRD) tracking in breast cancer, offering a surrogate of molecular relapse with clinically meaningful lead times to radiological relapse^10–13^. Such technological advancements have created opportunities for deployment in the metastatic setting, where a higher sensitivity and specificity of these assays for ctDNA monitoring could enable innovative approaches to clinical monitoring or support interventional treatment strategies. However, it remains unclear which parameters of longitudinal ctDNA assessment (i.e., molecular response and progression), optimal assessment timing, and stratification cutoffs are most relevant in predicting radiographic responses and survival outcomes. To the best of our knowledge, no studies to date have utilized tumor-informed ctDNA approaches in the context of patients with metastatic breast cancer receiving standard systemic therapy.

The goal of the present investigation was to evaluate a highly sensitive tumor-informed ctDNA assay, with a test limit of detection (LoD95) of 0.001% variant allele frequency (VAF), in HR+/HER2− MBC patients receiving standard of care ET and CDK4/6i. We focused on elucidating its potential as a prognostic biomarker for treatment response and clinical outcomes that could be used to optimize clinical follow-up, treatment decisions, and clinical trial design.

## Results

### Patient characteristics and samples

**Fig. 1:**
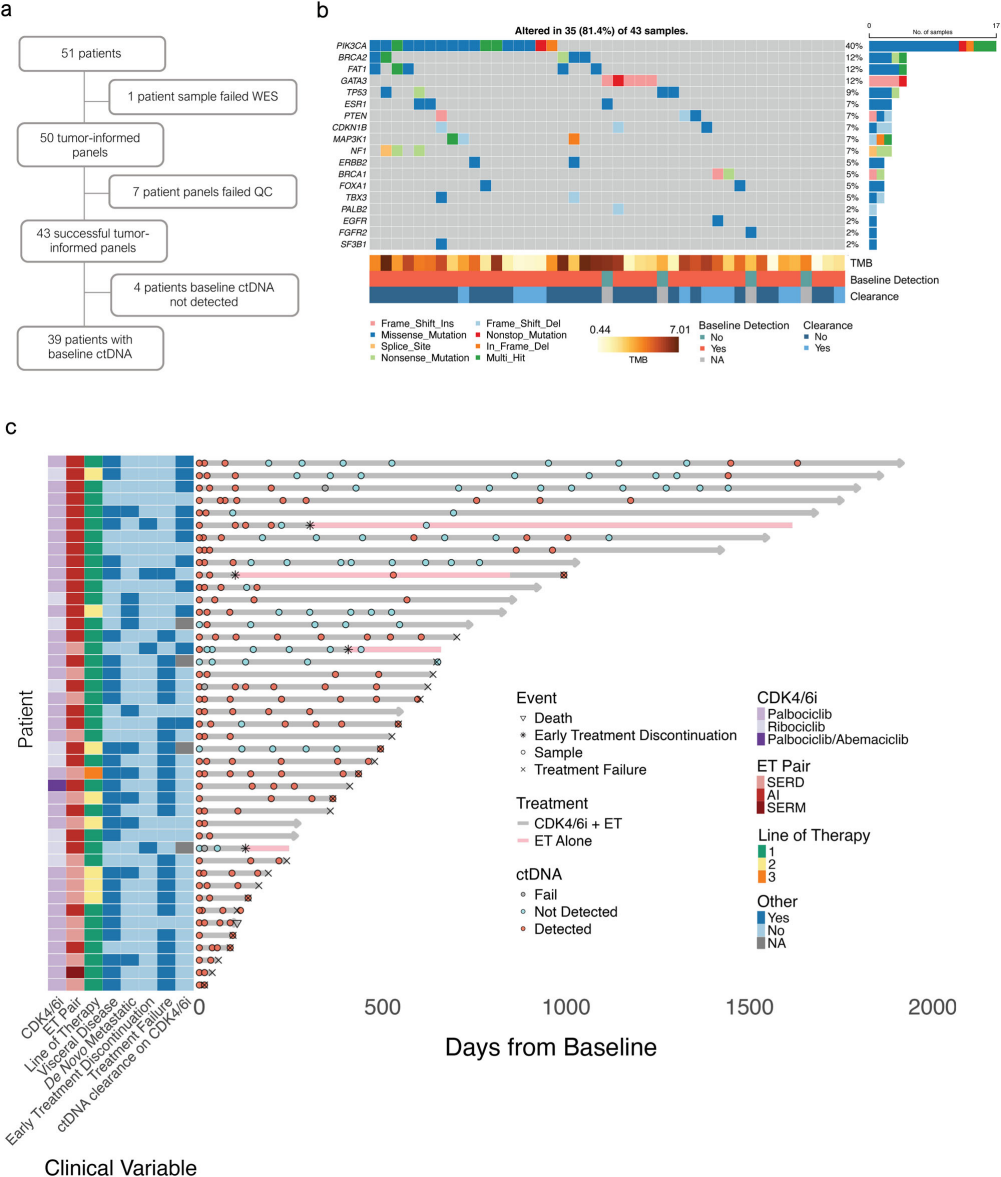
Patient characteristics and samples. **a** Flow diagram. **b** Oncoprint illustrating key genomic characteristics from whole exome sequencing of archival tumor tissue. **c** Swimmer plot depicting ctDNA timepoints, key clinical characteristics and outcomes. AI aromatase inhibitor, CDK4/6i cyclin dependent kinase 4 and 6 inhibitor, ctDNA circulating tumor DNA, ET endocrine therapy, QC quality control, SERD selective estrogen receptor degrader, SERM selective estrogen receptor modulator, TMB tumor mutational burden, WES whole exome sequencing.

A total of 51 HR+/HER2− MBC patients receiving standard ET and CDK4/6i were enrolled in this prospective observational cohort from July 2018 to April 2023 (Fig. 1a and Supplementary Fig. 1a). Over a median follow-up of 28.0 months (range 1.6–66.0), 279 longitudinal blood samples were collected, with a median of 5 (2–16) per patient (Supplementary Table 1). Tumor-informed panels were constructed successfully for 43 patients with 250 samples total, who composed the analytical cohort (Fig. 1a; Supplementary Tables 2, 3). For those 8 patients without a panel successfully designed, one was due to the failure of whole exome sequencing (WES), and 7 were due to too few target somatic variants remaining after a panel QC step (that filters for variants of germline and/or CHIP origin) to allow for optimal performance of the assay, which was developed for MRD detection. Detailed baseline patient and tumor characteristics, along with treatment trajectories, are reported in Table 1, Fig. 1b, c, Supplementary Table 4, and Supplementary Fig. 1b, c. In the analytical cohort, most patients were postmenopausal (81%), had visceral disease (65%), and received first-line therapy (79%) with an aromatase inhibitor (AI; 63%) and palbociclib (79%) while on the study. Archival tissue WES identified a median of 162 somatic mutations per patient (39–623), resulting in a median tumor mutation burden (TMB) of 1.8 mutations per megabase (0.4–7.0) (Fig. 1b; Supplementary Table 5). Driver gene alterations clinically relevant in breast cancer or related to CDK4/6i or ET biology were identified, including mutations in *PIK3CA/AKT1/PTEN* in 19 (44.2%) patients, *BRCA1/BRCA2/PALB2* in 8 (18.6%) patients, *FAT1* in 5 (11.6%) patients, *TP53* in 4 (9.3%) patients, *ESR1* in 3 (7.0%) patients (all previously exposed to AI), and *ERBB2* in 2 (4.7%) patients. Archival tissue WES was used to design tumor-informed assays targeting a median of 48 (23–52) tumor-specific variants, where the archival tissue site of origin was breast in 79% of samples and the median time from tissue collection to baseline blood sampling was 1.72 years (−2.62–16.52) (Supplementary Fig. 1c; Supplementary Tables 2, 3). In our cohort, 4 patients discontinued the CDK4/6i for reasons other than disease progression and remained on ET alone (2 due to patient preference during the COVID pandemic; 1 due to recurrent infection; 1 due to liver toxicity). One additional patient was switched from palbociclib to abemaciclib due to recurrent neutropenia. One patient died due to causes unrelated to cancer. Treatment failure and death events during the study follow-up time were recorded in 24 (55.8%) and 21 (48.8%) of 43 patients, respectively (Supplementary Fig. 1d, e). Among patients experiencing an event, median time to treatment failure was 12.4 months (0.5–32.2) and median overall survival was 24.9 months (1.6–44.2). Eight (18.6%) patients experienced primary disease resistance, defined as disease progression within the first 6 months of therapy. A total of 234 standard of care staging CT scans were performed, with median interval between assessments of 3.8 months (0.5–14.5). RECIST v1.1 measurable disease was present at baseline in 27 (62.7%) patients (Supplementary Fig. 1d). In this group, a partial response was observed as best response in 19 (70.3%), and radiographic progression was recorded in 16 (59.3%) during the study follow-up, mostly due to progression of non-target lesions or appearance of new findings (12 of 16, 75.0%).

**Table 1:** Cohort characteristics

Cohort	All (*n* = 51)	Analytical cohort (*n* = 43)
**Characteristic**		
Age at diagnosis – median [range]	52.8 [36.5–81.7]	52.8 [36.5–81.7]
Age at study entry – median [range]	59.4 [38.1–88.2]	63.4 [38.1–88.2]
Age at study entry – no. (%)		
<40	1 (2)	1 (2)
40–50	6 (12)	5 (12)
50–60	18 (35)	11 (26)
>60	26 (51)	26 (60)
Sex – no. (%)		
Female	50 (98)	43 (100)
Male	1 (2)	0 (0)
Premenopausal – no. (%)	10 (20)	8 (19)
De novo metastatic – no. (%)	16 (31)	11 (26)
Disease sites – no. (%)		
Visceral	34 (67)	28 (65)
Bone-only	10 (20)	8 (19)
Line of therapy – no. (%)		
First	40 (78)	34 (79)
Second	9 (18)	8 (19)
Third or above	2 (4)	1 (2)
CDK4/6 inhibitor – no. (%)		
Palbociclib	38 (74)	34 (79)
Ribociclib	11 (22)	8 (19)
Abemaciclib	2 (4)	1 (2)
Endocrine therapy pair – no. (%)		
Aromatase inhibitor	34 (67)	27 (63)
SERD	16 (31)	15 (35)
SERM	1 (2)	1 (2)

The analytical cohort was composed of patients in whom personalized ctDNA panels were built successfully.

*CDK4/6* cyclin dependent kinase 4/6, *SERD* selective estrogen receptor degrader, *SERM* selective estrogen receptor modulator.

### Baseline tumor-informed ctDNA: clinicopathological, genomic, and prognostic associations

**Fig. 2:**
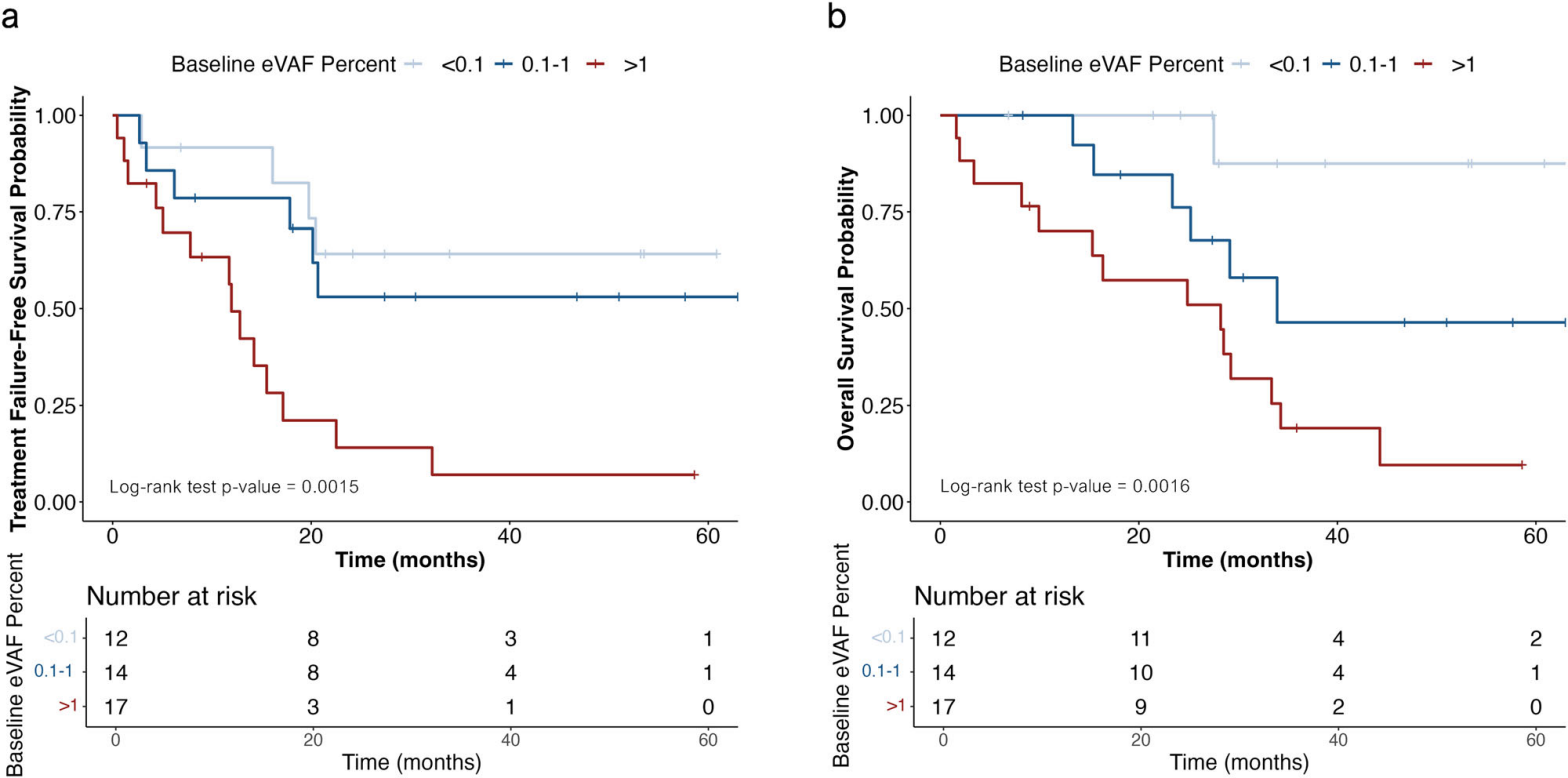
Baseline ctDNA. Time to treatment failure (**a**) and overall survival (**b**) according to baseline ctDNA levels.

ctDNA was detected at baseline in 39 (91%) of 43 patients with a median estimated variant allele fraction (eVAF_BL_) of 0.5% (0.006–17.9%). In 8 (21%) of 39 patients, eVAF_BL_ was <0.1%. eVAF_BL_ was significantly higher in patients with liver metastases (median eVAF 2.92% vs 0.32%, *p* = 0.02) but was not associated with other clinicopathological or genomic covariates in our cohort (e.g., bone-only disease, history of primary endocrine resistance, contemporality of archival tissue for panel generation, PI3K pathway alterations, DNA damage repair alterations, TMB) (Supplementary Fig. 2a). eVAF_BL_ did not significantly differ between patients with RECIST v1.1 measurable and non-measurable disease (*p* = 0.43); it was, however, positively correlated with the maximum diameter of target lesions as per RECIST v1.1 in patients with liver disease (rho 0.69, *p* < 0.01) but not in the overall population (rho 0.3, *p* = 0.13) (Supplementary Fig. 2b, c). In all patients, higher eVAF_BL_ predicted a shorter TTF (per % unit increase HR 1.15 CI 95% 1.06–1.24, *p* < 0.01) and OS (per % unit increase HR 1.17 CI 95% 1.09–1.26, *p* < 0.01), which remained statistically significant after adjusting for the presence of visceral disease at study entry (Fig. 2a–b; Supplementary Fig. 2d–g). In patients with RECIST v1.1 measurable disease, higher eVAF_BL_ was numerically associated with a lower likelihood of partial response (eVAF_BL_ 0.5% vs 5.0% for patients with and without response, respectively; *p* = 0.12).

Baseline ctDNA was not detected in 4 patients, whose clinical features are summarized in Supplementary Table6. These patients had bone and/or lung metastases, with two experiencing disease progression at data cutoff (Supplementary Fig.3a, b). One of them had detectable ctDNA at the treatment failure timepoint (TTF 16.1 months; eVAF 1.29%). The other patient, whose plasma remained ctDNA negative throughout, experienced disease progression leading to treatment failure at 20.5 months. Due to the discordance between ctDNA detection and clinical outcomes, and prompted by the complexities of panel design, an exploratory analysis was conducted for this patient. By refining the calling pipeline to focus on 4 variants best supported by QC, ctDNA became detectable at all timepoints, with 3 variants being consistently positive and VAFs tracking clinical evolution (Supplementary Fig. 3c). While encouraging, the exploratory nature of these findings warrants caution and excluded them from subsequent analyses.

### ctDNA monitoring: early kinetics and association with RECIST v1.1 metrics

**Fig. 3:**
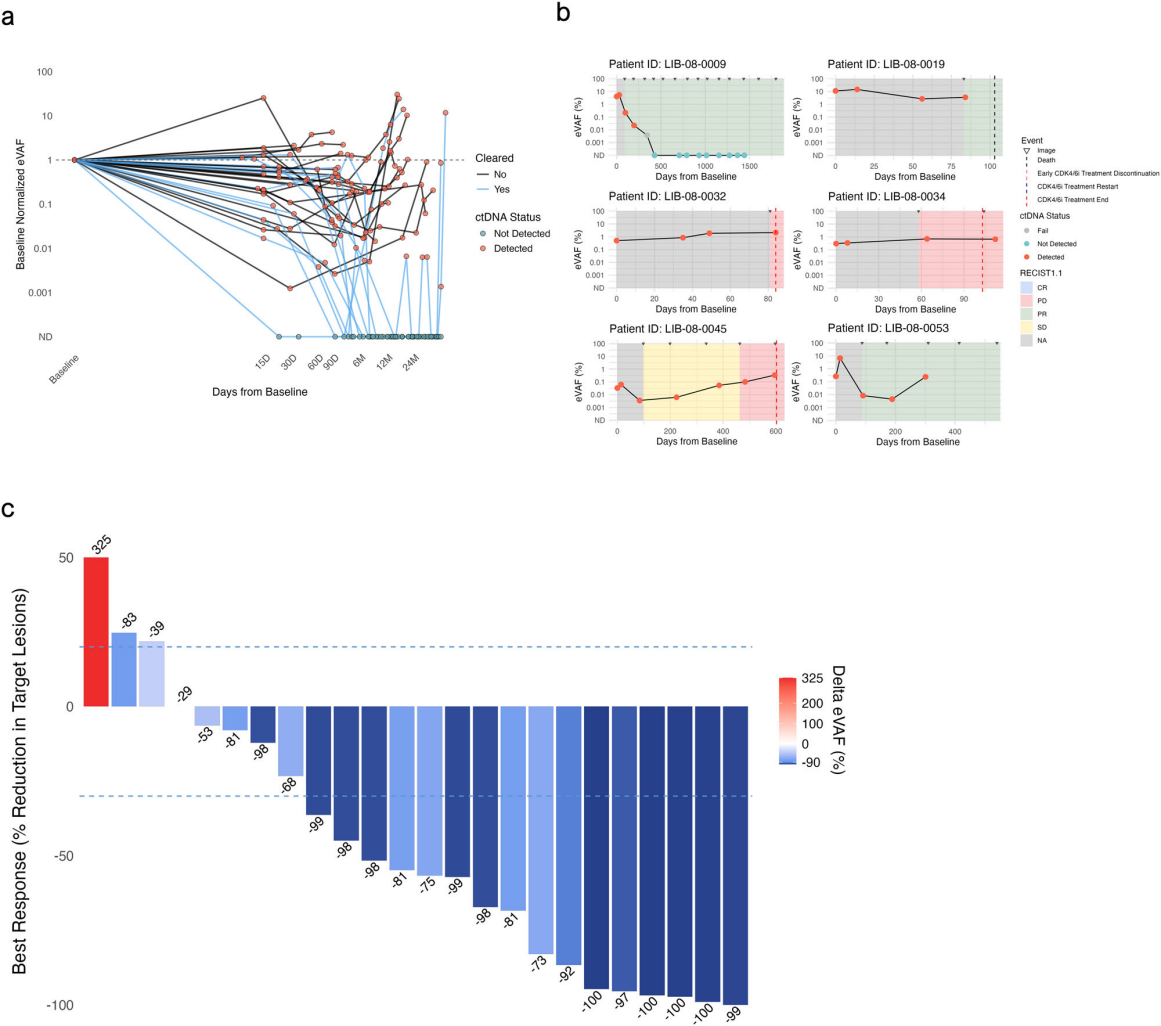
ctDNA monitoring. **a** ctDNA kinetics in patients with ctDNA detected at baseline until discontinuation or last follow-up. Failed samples are not shown. **b** Early ctDNA kinetics did not predict TTF (case vignettes). **c** Waterfall plot showcasing eVAF decrease from baseline and RECIST v1.1 target lesion change at best response. Dashed lines denote +20% and −30% thresholds for progressive disease and partial response as per RECIST v1.1. CR complete response, NA not applicable, PD progressive disease, PR partial response, SD stable disease.

ctDNA was detected in 174 (70.0%) of 250 total blood samples. The median eVAF for all timepoints was 0.29% (0.0008–34.94%), with 67 (39%) and 28 (16%) positive timepoints having an eVAF <0.1% and <0.01%, respectively. eVAF significantly decreased during treatment and increased upon radiographical progression leading to treatment failure (median eVAF 0.11% [0.0008–17.79%] and median eVAF 1.43% [0.01–34.94%], respectively; *p* < 0.01). Most patients (77.8%) had early (i.e., within the first cycle of 28 days) eVAF decreases below baseline, and 88.9% of patients showed such decreases before the first restaging scan (median time to first scan 2.9 months, 0.8–5.12) (Fig. 3a, Supplementary Fig. 3a, b). Early (i.e., ≤29 days) rises above baseline were observed in 6 (13.9%) patients and were not associated with worse outcomes; three of these patients experienced prolonged TTF, with their eVAF spike shortly followed by a decrease below baseline (Fig. 3b**;** Supplementary Fig. 3d). Moreover, only 2 of the 8 patients experiencing primary disease progression had an early eVAF increase (Supplementary Fig. 3a, b). In 27 patients with RECIST v1.1 measurable disease, ctDNA dynamics captured target lesion measurement changes, as reflected by a moderate correlation of ∆eVAF and ∆diameters at the time of best response (rho 0.69, *p* < 0.01) (Fig. 3c; Supplementary Fig. 3e). Two (7.4%) patients showed discrepancies, with ctDNA reductions from baseline but progressive disease leading to treatment discontinuation (Supplementary Fig. 3f). Notably, for one of these patients, the ctDNA sample collected at the time of treatment failure revealed a 6-fold increase in eVAF compared to the preceding sample.

### ctDNA monitoring: defining molecular response

**Fig. 4:**
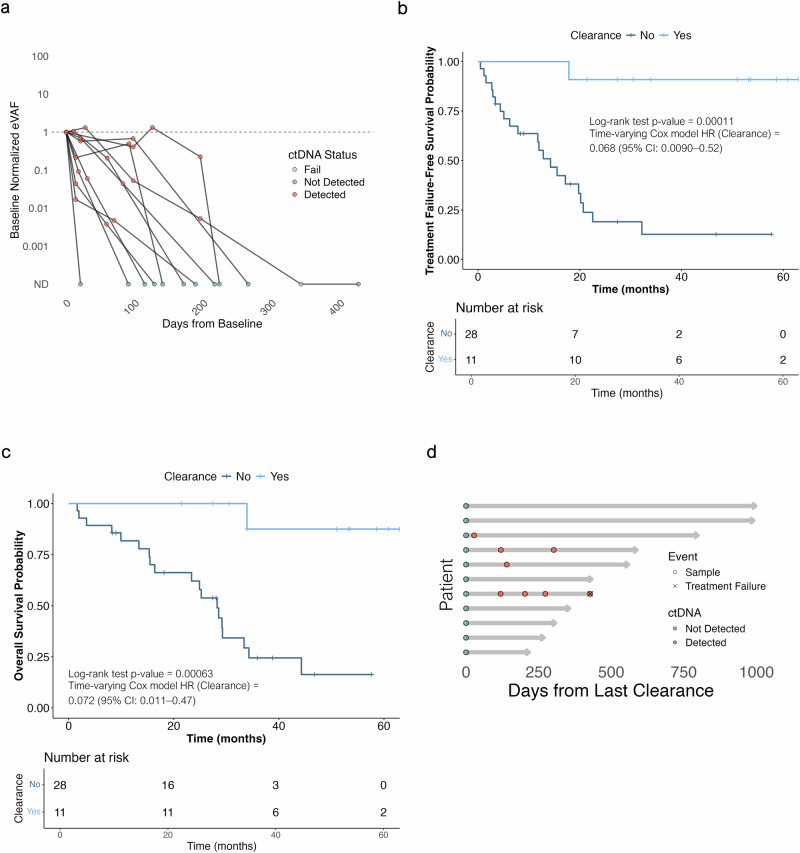
ctDNA monitoring: defining molecular response. **a** ctDNA kinetics in patients with ctDNA clearance. **b** Time to treatment failure and **c** overall survival according to ctDNA clearance, defined retrospectively based on the entire CDK4/6i treatment follow-up period. **d** Swimmer plot showing the time elapsed from the last cleared ctDNA timepoint to treatment failure or last study follow-up.

In our cohort, the lowest eVAF in a sample called as having ctDNA detected was 0.0008%. ctDNA clearance was observed in 11 (28%) of the 39 patients who had detectable ctDNA at baseline and the median time to its first occurrence was 5.7 months (0.5–13.5) (Fig. 4a). The clinicopathological characteristics of this cohort can be found in Supplementary Table 7. Patients with ctDNA clearance had longer TTF (time-varying Cox HR 0.068, CI 95% 0.009–0.52, *p* < 0.01). Median TTF was not reached in patients with cleared ctDNA, compared to 14.3 months in those without clearance. Treatment failure rates were 0% vs 45% at the 1-year landmark, and 12% vs 81%, at the 2–year landmark, respectively. Similarly, ctDNA clearance predicted a longer OS (time-varying Cox HR 0.072, CI 95% 0.011–0.47, *p* < 0.01), with median OS not reached in patients with cleared ctDNA, compared to 29.3 months in those without clearance (Fig. 4b–c; Supplementary Fig. 4a, b). To further account for the time interval before ctDNA clearance and potential immortal bias in survival outcomes associated with its late occurrence, landmark analysis at 12 months was performed and showed consistent results (Supplementary Fig. 4c, d). Clearance was more common in patients with eVAF_BL_ < 0.1% (*p* < 0.01), in those receiving AI while on study (*p* = 0.01), and in those with radiographic responses (*p* = 0.06); however, association trends were not observed with other relevant clinical covariates such as disease sites, therapy line, type of CDK4/6i, or history of primary endocrine resistance (Supplementary Fig. 4e). Notably, among patients who had a partial radiographic response (*n* = 17), those with ctDNA clearance (*n* = 7; 41%) had significantly longer TTF (HR 0.08 CI 95% 0.01–0.65, *p* = 0.019) and OS (HR 0.08 CI 95% 0.01–0.71, *p* = 0.023) (Supplementary Fig. 4f–g). At a median follow-up of 50.9 months (21.4–62.9), only one of 11 patients with ctDNA clearance had experienced treatment failure (TTF 17.9 months). The median time elapsed from the last cleared timepoint to treatment failure or last follow-up was 14.3 months (7.2–28.9) (Fig. 4d). In 8 of the 11 patients, on treatment ctDNA clearance was observed at two consecutive timepoints (Supplementary Fig. 3a, b). None of these patients with ctDNA cleared on consecutive sampling experienced treatment failure after a median follow-up of 52.3 months (50.9–62.9), with a median lead time from ctDNA clearance to the last follow-up of 13.0 months (7.2–19.8).

Considering less stringent definitions for molecular response, an eVAF reduction of more than 90% from baseline (eVAF_<10%_) was observed in 24 (61.5%) patients, took a median time of 2.8 months (0.5–31.7), and was associated with longer TTF (HR 0.21 CI 95% 0.09–0.51, *p* = 0.0002) and OS (HR 0.24 CI 95% 0.09–0.60, *p* = 0.001) (Supplementary Fig. 4h–k). The median interval from the last eVAF_<10%_ timepoint to treatment failure or last follow-up was 11.0 months (1.9–32.8), with median of 7.5 months to treatment failure among patients who experienced the event while on study, and 16.7 months to last follow-up in ongoing patients. When matched on-treatment ctDNA timepoints and restaging CT scans were compared (up to +/−30 days; 113 samples from 36 patients), all plasma timepoints with ctDNA clearance or eVAF_<10%_ showed either response or stability in corresponding CT scans both as per RECIST v1.1 and as appraised by the reporting clinical radiologist.

### ctDNA monitoring: exploring definitions of molecular progression

Treatment failure occurred in 24 (55.8%) patients during the follow-up period, 17 (70.8%) of whom had a ctDNA rise at any timepoint during therapy. To characterize the test performance of eVAF increase as a definition of molecular progression, we analyzed matched ctDNA timepoints and restaging CT scans. We excluded CT scans showing early bone pseudoprogression (*n* = 3)—where new bone lesions reflected osteoblastic bone healing with clinical benefit and concurrent or subsequent treatment response; all matched samples showing eVAF decreases from baseline—and patients with undetectable ctDNA at baseline (*n* = 4).

**Fig. 5:**
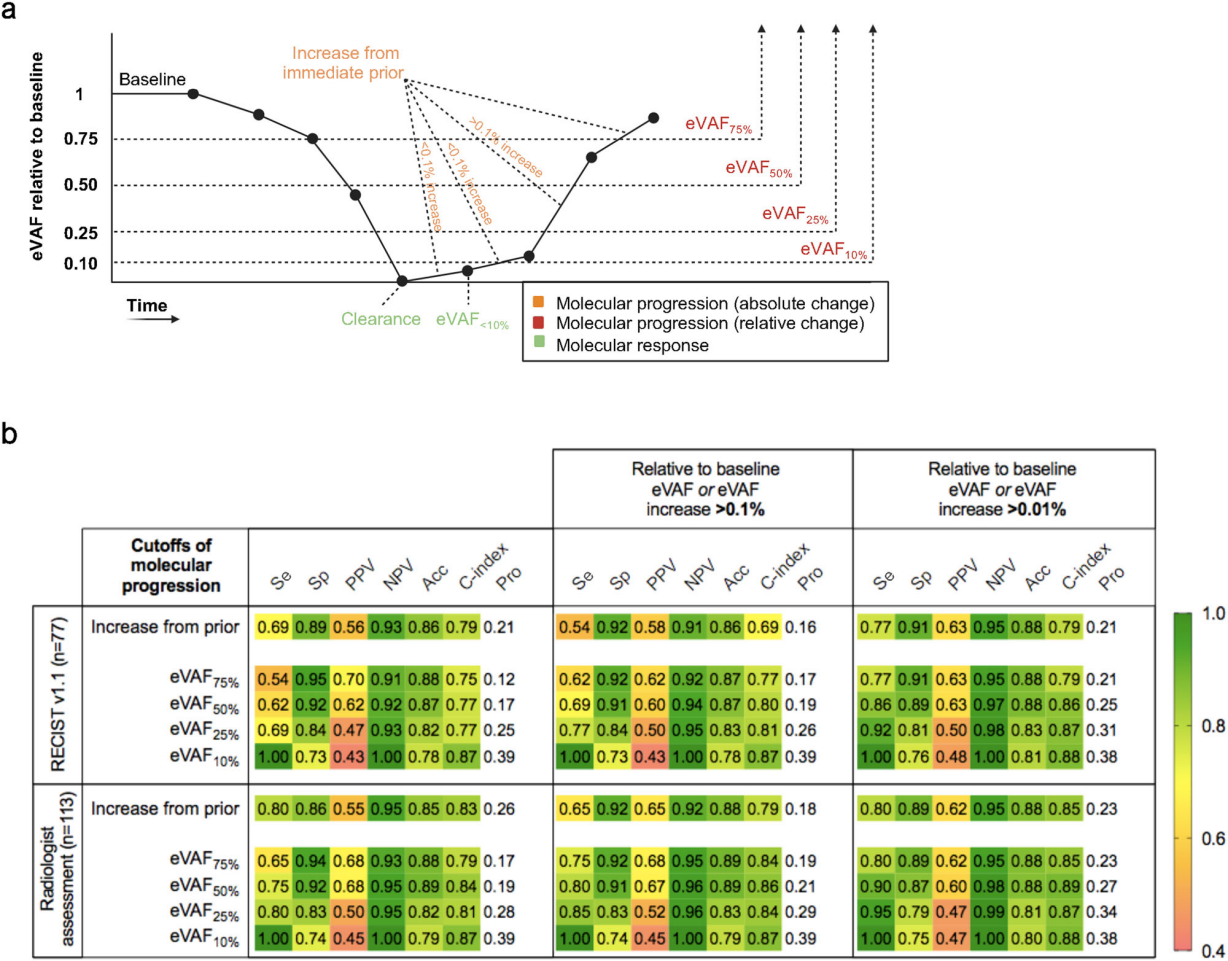
ctDNA monitoring: defining molecular progression. **a** ctDNA dynamics over time and different exploratory definitions of molecular progression. **b** Test performance of different definitions of molecular progression. Performance characteristics were analyzed comparing matched on-treatment ctDNA timepoints and restaging CT scans, where the time interval between them could be up to +/−30 days. Se sensitivity, Sp specificity, PPV positive predictive value, NPV negative predictive value, Acc accuracy, Pro proportion.

Any eVAF rise from the immediate prior sample (26% of samples) demonstrated a sensitivity of 80%, specificity of 86%, PPV of 55%, and NPV of 95%, to detect radiographic progression as adjudicated by the clinical radiologist (C-index 0.83) (Fig. 5a, b). By restricting the threshold for molecular progression to eVAF rises >0.01% (23% of samples), in order to control for small changes that could be attributed to technical artifacts or biological variation, specificity rose to 89% and PPV to 62% (C-index 0.85). Among the false positive cases (10 timepoints from 7 patients) where molecular progression was detected without concurrent radiological progression, the median eVAF increase was 0.20% (0.02–7.51) and 8 of 10 timepoints were classified as stable disease. After a median follow-up of 58.9 months (18.6–65.0), 3 of 7 patients had experienced treatment failure and the lead time of molecular progression was 9.8 months (4.6–13.9), while the median time to the last follow-up in ongoing patients was 9.7 months (8.3–19.2).

To further characterize molecular progression, we investigated the performance characteristics of several exploratory definitions based on different thresholds capturing the relative change from eVAF_BL_ (i.e., absence of decrease or decrease of less than 25% (eVAF_75%_), 50% (eVAF_50%_), 75% (eVAF_25%_), and 90% (eVAF_10%_)) (Fig. 5a, b). An eVAF_75%_ (12% of samples) showed the highest test accuracy (88%) to detect imaging disease progression, with a PPV of 70%, a NPV of 91%, a sensitivity of 54%, and a specificity of 95%, in the RECIST v1.1 measurable population (C-index 0.75). Of note, one patient who had an eVAF_75%_ without concurrent RECIST v1.1 progression, where a target lesion cervical lymph node went from 25 to 26 mm, was discontinued due to clinical progression with local infiltration of adjacent structures and worsening bone disease which were not clearly captured in the RECIST v1.1 assessment. In the full cohort including RECIST v1.1 measurable and non-measurable patients, a PPV of 68%, a NPV of 93%, a sensitivity of 65%, and a specificity of 94% were observed (C-index 0.79). When considering all timepoints in patients who experienced treatment failure during the study follow-up, eVAF_75%_ showed a lead time to treatment failure of 5.1 months (IQR 3.3–8.3). Conversely, eVAF_10%_ (39% of samples) showed the maximum sensitivity (100%) to capture concurrent progression in imaging, with a specificity of 73%, PPV of 43% and NPV of 100%, in the RECIST v1.1 measurable population (C-index 0.87), and a sensitivity of 100%, specificity of 74%, PPV of 45%, and NPV of 100%, in the complete cohort (C-index 0.87). eVAF_10%_ showed a lead time to treatment failure of 7.3 months (IQR 3.9–12.4).

To better elucidate the importance of absolute eVAF changes in these cutoffs relative to baseline, we calculated the performance characteristics of the combined absolute and relative cutoffs (Fig. 5b). The definition of molecular progression as any eVAF rise >0.01% from the immediate prior and/or an eVAF_50%_ (25% of samples) rendered the highest accuracy (88%) with a sensitivity of 86%, specificity of 89%, PPV of 63%, and NPV of 97% in the RECIST v1.1 measurable population (C-index 0.86), and a sensitivity of 90%, specificity of 87%, PPV of 60%, and NPV of 98% in the complete cohort (C-index 0.89). The combination of absolute and relative changes increased the test sensitivity and NPV but in turn resulted in more false-positive timepoints.

### Simulating plasma-triggered imaging follow-up strategies

Various strategies for ctDNA-informed assessment could be formulated from these data. One reasonable approach could consist of plasma-first longitudinal monitoring for patients having a molecular response (i.e., ctDNA clearance or eVAF_<10%_), with imaging reserved for confirmation of molecular progression. Such an approach would have resulted in no missed cases of progressive disease in our cohort, where 69/113 (61.1%) samples from 21/36 (58.3%) patients met criteria of molecular response among cases with matched on-treatment ctDNA timepoints and CT scans. Importantly, this definition would have performed equally for patients with RECIST v1.1 measurable and non-measurable disease. By focusing imaging on cases without molecular response, this approach could reduce the reliance on routine follow-up CT scans. Specifically, up to 61.1% of follow-up imaging tests and/or associated clinic visits for assessment of disease response might have been avoided in this cohort with blood-first monitoring. This conservative estimate leverages the 100% NPV of molecular response in predicting imaging progression in our matched dataset.

Complementarily, molecular progression metrics such as eVAF_75%_—observed in 26/207 on-treatment samples (13%) from 18/43 patients (42%) in the analytical cohort—, with a PPV of 70% and relatively short lead time to treatment failure (median 5.1 months, IQR 3.3–8.3), could trigger urgent imaging to rule out progressive disease. Further, we showed that patients who presented an eVAF_10%_ at some point during treatment—observed in 71/207 on-treatment samples (34%) from 31/43 patients (72%)—had a shorter TTF and OS, with a median lead time to treatment failure of 7.3 months (IQR 3.9–12.4). This may represent an opportunity for consideration of early treatment changes and intensified imaging follow-up.

## Discussion

Longitudinal ctDNA analysis using a high-sensitivity tumor-informed assay in this study revealed valuable insights into ctDNA dynamics and timing in patients with HR+/HER2−negative MBC undergoing standard endocrine and CDK4/6i therapy. Our cohort included a homogenous population that mirrored the main clinicopathological and genomic characteristics of published studies^4,5,14–20^. Forty-nine of the 51 patients were treated as first- or second-line during the study, only 3 patients were pre-treated with chemotherapy in the metastatic setting, and the follow-up time was sufficiently prolonged to capture treatment failure and OS events in 50% and 40% of the population, respectively. Treatment choice was dependent on the physician’s and patient’s decision, and CT scan frequency followed standard of care and was generally consistent among participants. We believe that these characteristics make our population representative of real-world practice.

Tumor-informed panels were generated successfully in 43 of 51 patients, with the main attrition being due to 7 patients having an insufficient number of target somatic mutations (<8) remaining after the panel QC step. At baseline, the test showed a sensitivity of 91%, which did not significantly differ between patients with RECIST v1.1 measurable and non-measurable disease. Higher eVAF_BL_ levels were prognostic and associated with the presence of liver disease, likely reflecting increased ctDNA shredding, both observations being consistent with prior studies in breast and other tumors^21–23^. During treatment, sensitivity was 67%, the lowest detected eVAF was 0.0008%, and changes in ctDNA levels and tumor size were moderately correlated. Taken together, these capabilities may offer an advantage over most tumor-agnostic blood-based NGS panels, enabling detection of subtle ctDNA changes in more patients during treatment^24,25^. While our results reflect the performance of the assay used—which required a minimum of 8 validated somatic mutations after the QC, as intended for MRD detection—it is conceivable that a less stringent mutation threshold could provide reasonable performance in the setting of advanced disease while reducing attrition.

We aimed to define exploratory, clinically meaningful cutoffs of molecular response and progression. ctDNA clearance, subject to the test limit of detection (LoD95) of 0.001%, was observed in 11 (28%) patients, first occurred at a median of 5.7 month, and was associated with longer TTF and OS^26^. Treatment with an AI while on study was associated with a higher likelihood of ctDNA clearance, likely implying earlier line of therapy and endocrine sensitivity, although the limited sample size permitted only a descriptive analysis. At a median 50.9 months (21.4–62.9) of follow-up, only one of 11 patients had experienced treatment failure, while none of the 8 patients exhibiting ≥2 consecutive cleared samples had had treatment failure after a median follow-up of 52.3 months (50.9–62.9). The favorable prognosis associated with ctDNA clearance was also observed among patients with RECIST v1.1 partial response. Similar results were observed with eVAF_<10%_. Importantly, no matched imaging assessments showed disease progression when clearance or eVAF_<10%_ were observed. The median intervals from the last cleared and eVAF_<10%_ samples to treatment failure or last study follow-up were 14.3 months (7.2–28.9) and 11.9 months (1.9–32.8), respectively. These data highlight the potential for plasma-first monitoring in patients with significant ctDNA decreases, which may permit reduction in the intensity of CT scans and clinic visits to evaluate treatment response. This molecular response metric could also pave the way for potential ctDNA-guided adaptive treatment de-escalation strategies^1,2,27^.

We next assessed ctDNA cutoffs to establish a molecular progression parameter in our cohort. Using any absolute ctDNA rise as a marker of molecular progression captured 80% of radiological progressions and achieved a 95% NPV. However, this approach showed a limited 55% PPV, making it a limited surrogate for imaging follow-up, even when restricting to absolute increases >0.01% to account for technical artifacts and biological variability. Conversely, eVAF_75%_ traded off sensitivity (54%) for the highest PPV (70%) and provided a median lead time to treatment failure of 5.1 months but was identified in only 12% of samples. By combining absolute and relative eVAF dynamics in the definition of molecular progression, we observed that eVAF_50%_ and/or any rise >0.01% from the immediate prior sample had the highest discriminatory ability and rendered an 86% sensitivity and 63% PPV, with this molecular progression metric being observed in 25% of samples. Although these definitions of molecular progression resulted in a significant false-positive rate, they could still be employed reliably to prompt reflex imaging during plasma-first follow-up or to inform treatment escalation in clinical trials. Regarding the latter, most ctDNA-guided clinical trials have thus far been limited to MRD detection in the early setting, with the PADA–1 and SERENA–6 trials—where treatment escalation is based on the emergence of *ESR1* mutant copies—serving as the only prominent examples in MBC^28–34^. While this approach has yielded promising results for ET substitution when rising *ESR1* mutations are detected, such a focused monitoring and intervention strategy accepts that most patients will not benefit, where the prevalence of *ESR1* mutations is ~40%.

The consideration of molecular response and progression biomarkers could facilitate a variety of plasma-first monitoring strategies. By using some of the exploratory cutoffs from our study (i.e., ctDNA clearance or eVAF_<10%_ as molecular response), approximately two thirds of CT scans and/or associated clinic visits for assessment of disease status could have been avoided. Regarding imaging follow-up, this could follow standard of care indication with eVAF_10%_ and be prioritized with eVAF_75%_, where PPV for radiological progression was highest. Thus, this approach has the potential to simplify follow-up for a substantial proportion of patients and, with the projected reductions in the cost of ctDNA assays, further alleviate financial and logistical burdens on patients, providers, and health systems.

Furthermore, potential opportunities arise for drug development in this setting where high sensitivity ctDNA monitoring may provide early molecular surrogates of prolonged efficacy, guide escalation and de-escalation strategies, and inform about the optimal timing for and interpretation of blood-based driver-focused NGS testing. In this regard, ctDNA-guided trials, so far employing tumor-agnostic NGS or PCR, have shown the clinical utility of baseline blood-based genomic characterization, as well as of early surrogates of response or progression, to achieve improved survival outcomes^33–35^. For instance, patients with molecular response could be allocated to intermittent CDK4/6i schedules, potentially delaying the emergence of resistant clones and extending treatment efficacy. A similar strategy, based on ctDNA-guided tyrosine kinase inhibitor break, has been evaluated in advanced non-small cell lung cancer with promising results^27^.

For the population with insufficient ctDNA reductions (i.e., eVAF_10%_), who exhibit worse prognosis, we may conceive clinical trials where patients are randomized to switch the ET or CDK4/6i component and/or add another agent. The allocation to this agent would ideally be guided by contemporaneous blood-based driver-focused NGS testing. In this regard, the higher sensitivity of the tumor-informed approach could accurately quantify tumor fraction, aiding the deployment or interpretation of tumor-agnostic NGS panels targeting clinically actionable gene alterations conveying resistance or sensitivity to therapies. Overall, by achieving a higher sensitivity of ctDNA detection during the course of treatment, tumor-informed approaches can expand the application of ctDNA monitoring, detect subtle ctDNA dynamics, and provide more robust biomarkers of response and progression.

Our study has several limitations. First, due to the ctDNA technique specifications, a tumor tissue sample is required for personalized panel generation, thus restricting the eligible population to patients with available tumor tissue material. It is possible that with optimized sampling or pathology workflows, such a limitation could be overcome. Second, recruitment and sample collection occurred during the COVID-19 pandemic, an event that influenced the timing of sample collection due to measures to reduce hospital visits to mitigate the risk of virus spread. As a consequence of this, the time interval between ctDNA sample collection and CT scans was not always aligned in our cohort. This impacted the characterization of molecular progression and associated lead times to treatment failure. Third, the relatively small sample size limited our ability to identify statistically significant associations, perform repeated measures analysis, and build logistic models; thus, our results must be interpreted as exploratory, cohort-dependent and hypothesis-generating, requiring validation in larger prospective studies. Fourth, the vast majority of our patients received palbociclib, which was the most commonly prescribed CDK4/6i at the time the cohort was accrued, thereby limiting the generalization of our results to patients treated with ribociclib or abemaciclib. Finally, with regard to the modeled blood-first monitoring approaches, we recognize that CT scans and clinic visits provide rich and nuanced information about the patient’s experience and disease evolution that cannot be fully captured with a ctDNA-based biomarker. ctDNA monitoring would be integrated in a comprehensive assessment of the patient’s journey, working as an early molecular surrogate of response and prognosis, and permitting baseline risk stratification and potential real-time risk-adapted treatment strategies.

In conclusion, we showed that tumor-informed ctDNA testing offers high sensitivity monitoring in patients with MBC receiving standard of care ET and CDK4/6i combinations. Substantial ctDNA reductions (i.e., molecular response: ctDNA clearance or eVAF_<10%_) while on treatment were associated with prolonged treatment benefit and identified patients with very low risk for radiological progression within a clinically meaningful interval, thus suggesting the opportunity for plasma-first follow-up strategies. Conversely, ctDNA rises or insufficient ctDNA reductions (i.e., molecular progression: eVAF_10%_) were associated with shorter time to treatment failure. Such plasma-based parameters and strategies have relevance to other treatment contexts (i.e., subtypes, therapies) but the specifics of ctDNA dynamics and diagnostic performance may vary and require further assessment. Additional research is needed to characterize molecular metrics and reveal their full potential in personalized treatment decision-making, follow-up strategies, and clinical trial design.

## Methods

### Patient recruitment

Patients diagnosed with HR+/HER2− MBC receiving standard of care ET in combination with CDK4/6i at the Princess Margaret Cancer Centre were prospectively enrolled for serial blood collection and banking from July 2018 to April 2023. Formal inclusion in the Liquid Biopsy Evaluation and Repository Development at Princess Margaret (LIBERATE) cohort (NCT03702309)—its matrix institutional program—began in August 2017, with the last participant included in this analysis enrolled in March 2023. Initiation of ET and CDK4/6i and choice of therapy was at the discretion of the treating physician in discussion with the patient. This study conforms to the REMARK guidelines for biomarker reporting and was approved by the University Health Network Research Ethics Board (REB#: 23-5111.1 and 18-6192.7). All participants provided informed consent prior to study enrollment and participation. ctDNA results were not returned to patients given the retrospective nature of these analyses. Standard clinical follow-up was delivered by treating physicians. Radiographic assessment was performed independently by radiologists as per standard of care and RECIST v1.1 assessment was performed by the investigators.

### Clinical specimens

Archival tumor tissue that was formalin-fixed and paraffin-embedded (FFPE) and obtained from routine diagnostic biopsies or surgical resections was selected to meet the specifications for assay generation. A review by a pathologist was conducted to evaluate the tumor’s overall cellular composition, ensuring that the sample contained 20–80% tumor cells. For nucleic acid extraction, either ten 10 µm slides or 2–3 cores, each measuring 1 mm², were utilized. Archival specimens were sent to NeoGenomics Laboratories, Inc. in Durham, North Carolina, where DNA extraction and WES were carried out according to previously outlined protocols^26,36^. Serial blood samples were collected at various points: at baseline, shortly after treatment initiation (on day 15 and/or cycle 2 day 1), approximately every three months during treatment alongside standard imaging, and at the time of disease progression leading to treatment cessation. Samples were collected using three Streck BCT tubes and underwent a double-spin process for separating plasma from the buffy coat prior to aliquoting and storage at −80 °C. For the assays, ~4 mL of plasma was obtained for each timepoint from which to isolate cell-free DNA (cfDNA).

### Clinical variables

Clinical, pathological, and demographic characteristics were collected via retrospective analysis of the electronic health record. Estrogen receptor and HER2 status were evaluated on each participant’s clinical diagnostic biopsy using ASCO/CAP guidelines as reported by the clinical pathologists^37,38^. Primary endocrine resistance was defined as relapse during the first 2 years of adjuvant ET or progressive disease within the first 6 months of first-line ET in the metastatic setting, and secondary resistance was defined as relapse while on adjuvant ET but after 2 years of treatment, relapse within 1 year of completing adjuvant ET, or progressive disease after 6 months of ET in the metastatic setting^39^. Survival endpoints included time to treatment failure (TTF) and overall survival (OS). TTF was defined as the time from ET and CDK4/6i start to the date of discontinuation of both agents for any reason. TTF was chosen as primary survival endpoint in this observational study to better reflect real-world clinical practice, particularly in a setting where treatment beyond RECIST v1.1 progression due to clinical benefit and patients with bone-only disease are common. Four patients discontinued the CDK4/6i due to various reasons other than disease progression and remained on ET alone. OS was defined as the time from ET and CDK4/6i start to the date of death of any cause.

### RaDaR sequencing analysis

The analysis of ctDNA was performed as detailed elsewhere^26^. In summary, FFPE tumor samples were sent to NeoGenomics in Durham, NC, for WES. The extraction of tumor DNA was performed using the Maxwell RSC DNA FFPE kit (Promega), and the quantity was assessed with the Quanti-IT dsDNA Broad Range assay (Invitrogen). For library preparation, the KAPA Hyper Prep kit (KAPA Biosystems) along with unique dual-indexed (UDI) adapters were employed, consistent with prior reported methodologies^10,26^. The IDT xGen Exome Research panel v1.0 was utilized to enrich the exomes of the pre-capture libraries, which were then analyzed for fragment size and quantification before pooling, normalization, and sequencing on the Illumina HiSeq4000 platform. The analysis of tumor-specific exome sequencing data was performed using a custom workflow that included processing fastq files, aligning them to the human genome (hg38), marking duplicates, and calling variants. An initial filtering step to remove germline variants was used, reliant on specific criteria based on information from public single nucleotide polymorphism (SNP) databases. Somatic variants identified in the tumor tissue through WES were prioritized using a proprietary algorithm, which facilitated the design of a patient-specific primer panel containing up to 48 primer pairs, each targeting at least one somatic variant. After assembling the primer panel, targeted sequencing was conducted on the tumor DNA and baseline buffy coat to confirm the presence of somatic variants. RaDaR assays utilized cfDNA extracted from plasma samples at various timepoints, in addition to a baseline buffy coat DNA control. This control sample served multiple functions: it allowed for the identification and removal of germline variants, excluded variants related to CHIP, and acted as a positive amplification control. Comprehensive details of the RaDaR assay workflow have been described in previous literature as well as the test limit of detection (LoD95) of 0.001%^10,26,40^. NeoGenomics was blinded to clinical outcomes at the time of sample analysis.

### Tumor WES analysis

To identify somatic genetic alterations for tumor molecular profiling, independent of the RaDaR ctDNA workflow, preprocessed WES BAM files from NeoGenomics were analyzed independently from the RaDaR ctDNA workflow as reported elsewhere^41^. Base quality score recalibration (BQSR) was completed using GATK (v3.8) tools and Samtools (v1.3.1) was used to sort and index the BAM files. Variant calling was performed using Mutect2 in tumor-only mode with orientation bias filtering and contamination estimation (GATK v4.1.8.1)^42–44^. Variants were called referencing the human genome (Homo sapiens assembly 38), targeting capture regions from the NeoGenomics panel, and using a germline resource and panel of normals for additional filtering. GATK’s FilterMutectCalls tool was used to further filter the variant call format (VCF) files. Annotations were added with VEP (v98) and then converted to mutation annotation format (MAF) via vcf2maf (v1.6.17)^45,46^. Custom scripts filtered variants according to these criteria: minimum depth of 100, variant allele frequency (VAF) above 5% to remove sequencing errors, VAF outside the ranges 40–60% or above 90% to exclude germline variants, specific mutation types (‘Missense_Mutation,’ ‘Nonsense_Mutation,’ ‘Frame_Shift_Ins,’ ‘Frame_Shift_Del,’ ‘Splice_Site,’ ‘In_Frame_Ins,’ ‘In_Frame_Del’), and allele frequency below 1% in the GNOMAD v.3.0 database^47^. Further filtering focused on known driver genes from the COSMIC Cancer Gene Census (v100)^48^. Oncoplot visualization was performed using maftools (v2.18.0) with RColorBrewer (v1.1-3) in R (v4.3.2)^49^.

### Statistical analysis

Patient baseline characteristics including clinical and ctDNA variables were compared using the Chi-square, Fisher’s exact, Wilcoxon rank sum or Kruskal Wallis tests where appropriate. Correlation between imaging measurements and quantified ctDNA levels was assessed using the Spearman coefficient. Survival outcomes were estimated using the Kaplan–Meier method, Mantel´s log-rank test, and Cox regression analysis. Participants who did not experience treatment failure or death of any cause by the last follow-up were censored at their last follow-up date. The TTF outcome was calculated in months from the date of treatment start to the date of the last on-treatment follow-up along with the event status on this date. The lead time of a given ctDNA sample was calculated as the interval between the date of sample collection and treatment failure. Diagnostic characteristics including sensitivity, specificity, positive predictive value (PPV), negative predictive value (NPV), accuracy, and C-index, were calculated to describe the discriminatory ability for each exploratory cutoff. The limited sample size and outcome events prevented us from building multivariable models. Generalized estimating equations were not calculated considering the impact of the timing of predictors (i.e., not accounting for the stronger impact of later timepoints on the final outcome nor modeling individual trajectories or temporal dynamics) as well as the limited sample size. Statistical analyses were performed using PRISM GraphPad v9.0 (San Diego, CA, USA) and an open-source statistical software R version 4.1.0 (R Core Team (2021), R Foundation for Statistical Computing, Vienna, Austria). All *p*-values are 2-sided, with *p* < 0.05 considered to indicate a statistically significant result. No multiple testing correction was applied. Median and range are shown unless otherwise indicated. Graphs were generated using R-studio, PRISM GraphPad (v9.0), and BioRender. Oncoplots and swimmer plots were created using R-studio.

## Supplementary information

Supplementary figures_

Supplementary tables_

## Data Availability

De-identified clinicopathological and processed genomic data that support the findings of this study are available in the supplementary material. Raw whole exome sequencing data that support the findings of this study have been deposited in the European Genome-Phenome Archive with the primary accession code EGAD50000001401.

## Code availability

Data analyses and data presentation were performed using open-source R (version 4.1.0) packages. Code can be obtained from the corresponding author upon reasonable request. Custom code underlying ctDNA signal classification from NeoGenomics is proprietary.
