# Supplementary material for: Personalized ctDNA monitoring in metastatic HR+/HER2− breast cancer patients during endocrine and CDK4/6 inhibitor therapy
Source: NPJ Breast Cancer. 2025 Jul 19;11:74. doi: 10.1038/s41523-025-00783-2 (PMC12276344; doi:10.1038/s41523-025-00783-2)

Supplementary figures

Title

Personalized ctDNA monitoring in metastatic HR+/HER2- breast cancer patients during endocrine and CDK4/6 inhibitor therapy

Authors

Jesús Fuentes-Antrás[1,2*†], Mitchell J. Elliott[1*], Sasha C. Main[3,4*], Philippe Echelard[5], Aaron Dou[1], Philippe L. Bedard[1], Eitan Amir[1], Michelle B. Nadler[1], Nicholas Meti[6], Nancy Gregorio[1], Elizabeth Shah[1], Emily Van de Laar[1], Celeste Yu[1], Yangqing Deng[7], Lisa Gates[8], Clodagh Murray[8], Christopher G. Smith[8], Amber Chevalier[8], Scott V. Bratman[3,4,9], Lillian L. Siu[1], Hal K. Berman[5], David W. Cescon[1†]

Affiliations

1. Division of Medical Oncology & Hematology, Department of Medicine, Princess Margaret Cancer Centre and University of Toronto, Toronto, ON, Canada
2. NEXT Oncology, Experimental Therapeutics Unit, Hospital Universitario Quirónsalud Madrid, Madrid, Spain
3. Department of Medical Biophysics, University of Toronto, Toronto, ON, Canada
4. Princess Margaret Cancer Centre, University Health Network, Toronto, ON, Canada
5. Department of Pathology and Laboratory Medicine, University Health Network, Toronto, ON, Canada
6. Gerald Bronfman Department of Oncology, St. Mary's Hospital Center, McGill University, Montreal, QC, Canada
7. Department of Statistics, University of Toronto, Toronto, ON, Canada
8. NeoGenomics, Babraham Research Campus, Cambridge, UK, Research Triangle Park, NC, USA
9. Department of Radiation Oncology, University of Toronto, Toronto, ON, Canada

*Equal contribution

[†]Corresponding author. Email: dave.cescon@uhn.ca, jesus.fuentesantras@uhn.ca

700 University Avenue, Suite 7-624, Toronto, Ontario, M5G1Z5 Canada

t. (416) 946 4501 ext. 2245; f. (416) 946-6546

Running title: Personalized ctDNA tracking in HR+/HER2- metastatic breast cancer

Suplemmentary Figure 1. (a) Study design. Personalized assay generation worklow. **(b)** Oncoprint showcasing key clinicopathological and treatment-related variables for the 43 patients with successful tumor-informed panels. **(c)** Site of origin and timing of archival tissue for personalized assay generation. **(d)** ctDNA timepoints and tumor response by RECIST v1.1. and **(e)** radiologist assessment. Abbreviations: CDK4/6i, cyclin dependent kinase 4 and 6 inhibitor; ctDNA, circulating tumor DNA; ET, endocrine therapy; FFPE, formalin-fixed paraffin-embedded; HER2, human epidermal receptor 2; HR, hormone receptor; IDC, invasive ductal carcinoma; ILC, invasive lobular carcinoma; MDLC, mixed ductal lobular carcinoma; SERD, selective estrogen receptor degrader; SERM, selective estrogen receptor modulator; WES, whole exome sequencing.

a

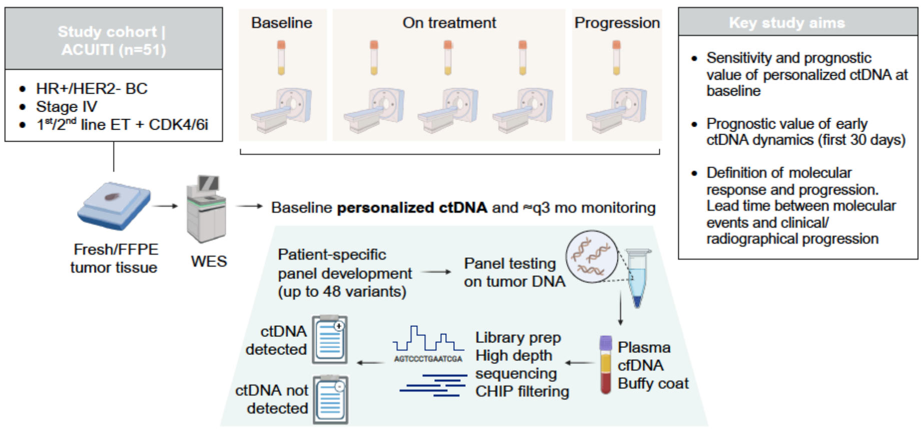

b

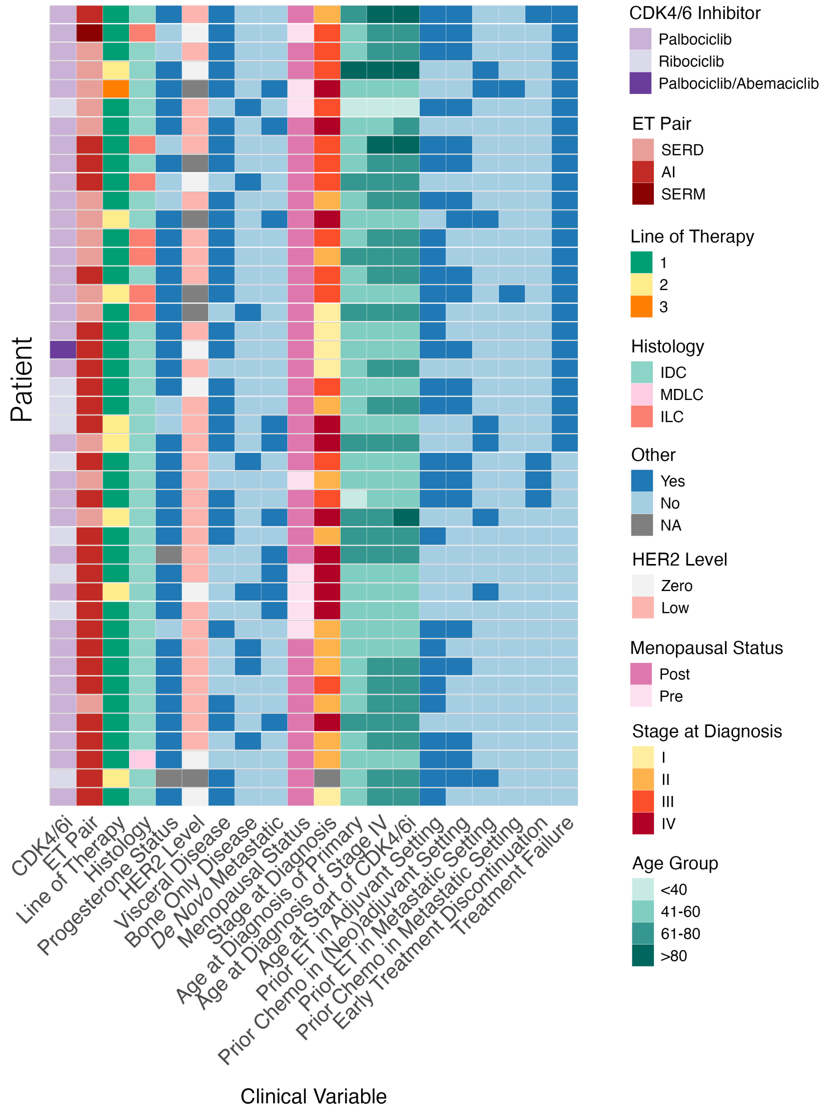

Suplemmentary Figure 1. (a) Study design. Personalized assay generation worklow. **(b)** Oncoprint showcasing key clinicopathological and treatment-related variables for the 43 patients with successful tumor-informed panels. **(c)** Site of origin and timing of archival tissue for personalized assay generation. **(d)** ctDNA timepoints and tumor response by RECIST v1.1. and **(e)** radiologist assessment. Abbreviations: CDK4/6i, cyclin dependent kinase 4 and 6 inhibitor; ctDNA, circulating tumor DNA; ET, endocrine therapy; FFPE, formalin-fixed paraffin-embedded; HER2, human epidermal receptor 2; HR, hormone receptor; IDC, invasive ductal carcinoma; ILC, invasive lobular carcinoma; MDLC, mixed ductal lobular carcinoma; SERD, selective estrogen receptor degrader; SERM, selective estrogen receptor modulator; WES, whole exome sequencing.

c

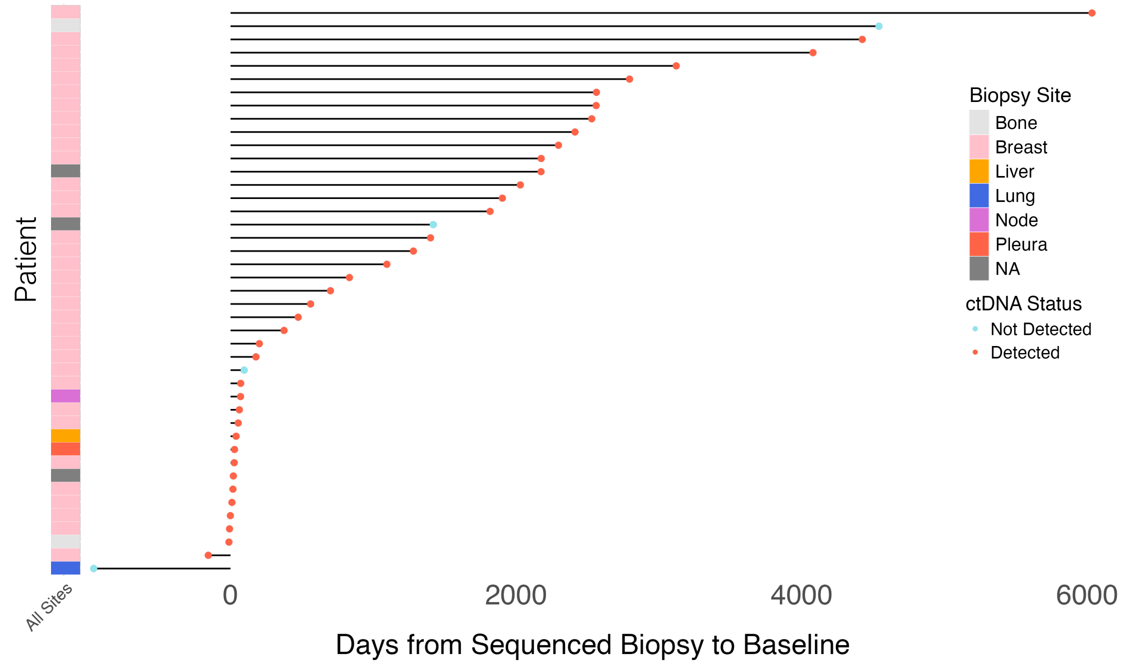

d

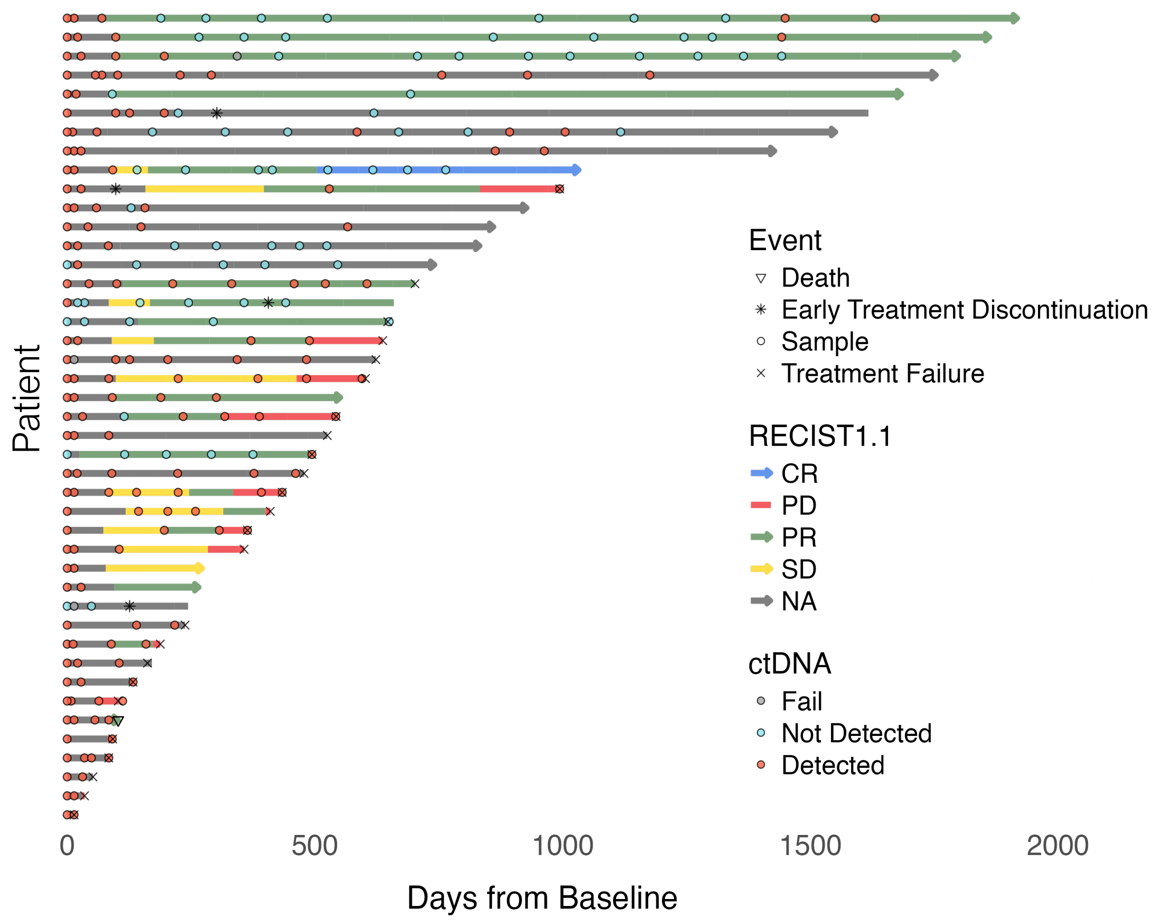

Suplemmentary Figure 1. **(a)** Study design. Personalized assay generation worklow. **(b)** Oncoprint showcasing key clinicopathological and treatment-related variables for the 43 patients with successful tumor-informed panels. **(c)** Site of origin and timing of archival tissue for personalized assay generation. **(d)** ctDNA timepoints and tumor response by RECIST v1.1. and **(e)** radiologist assessment. Abbreviations: CDK4/6i, cyclin dependent kinase 4 and 6 inhibitor; ctDNA, circulating tumor DNA; ET, endocrine therapy; FFPE, formalin-fixed paraffin-embedded; HER2, human epidermal receptor 2; HR, hormone receptor; IDC, invasive ductal carcinoma; ILC, invasive lobular carcinoma; MDLC, mixed ductal lobular carcinoma; SERD, selective estrogen receptor degrader; SERM, selective estrogen receptor modulator; WES, whole exome sequencing.

e

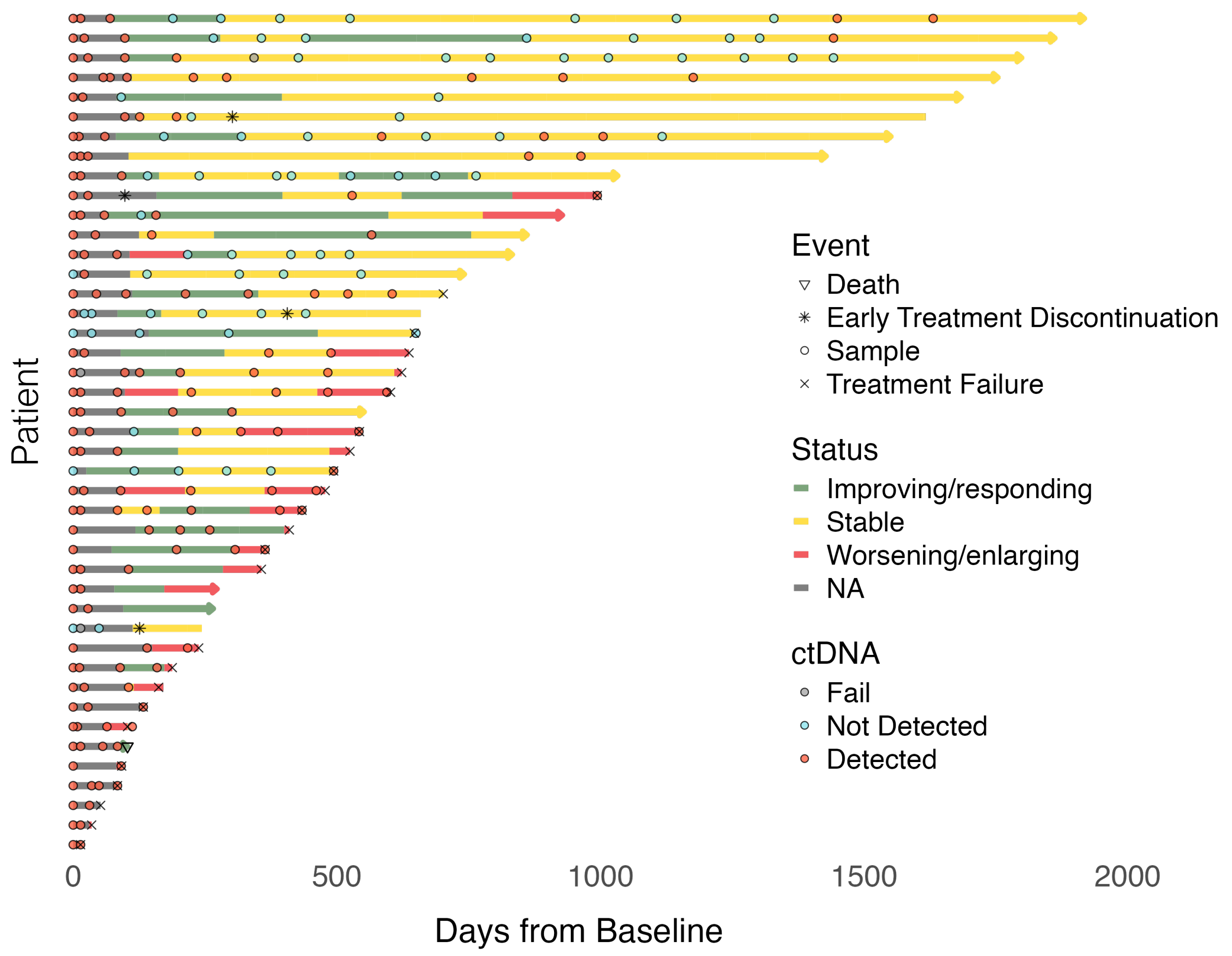

Supplementary Figure 2. (a) Clinicopathological and genomic associations with baseline eVAF, excluding failed samples. **(b)** Correlation of baseline eVAF with RECIST v1.1 target lesion measurements in the analytical cohort and **(c)** in patients with liver disease at baseline. **(d)** Time to treatment failure and **(e)** overall survival forest plots for univariable Cox models for baseline eVAF and key clinical variables including the presence of visceral disease, age at study entry, line of therapy, and ET pair on study. **(f)** Time to treatment failure and **(g)** overall survival forest plots for multivariable Cox models including baseline eVAF and the presence of visceral disease.

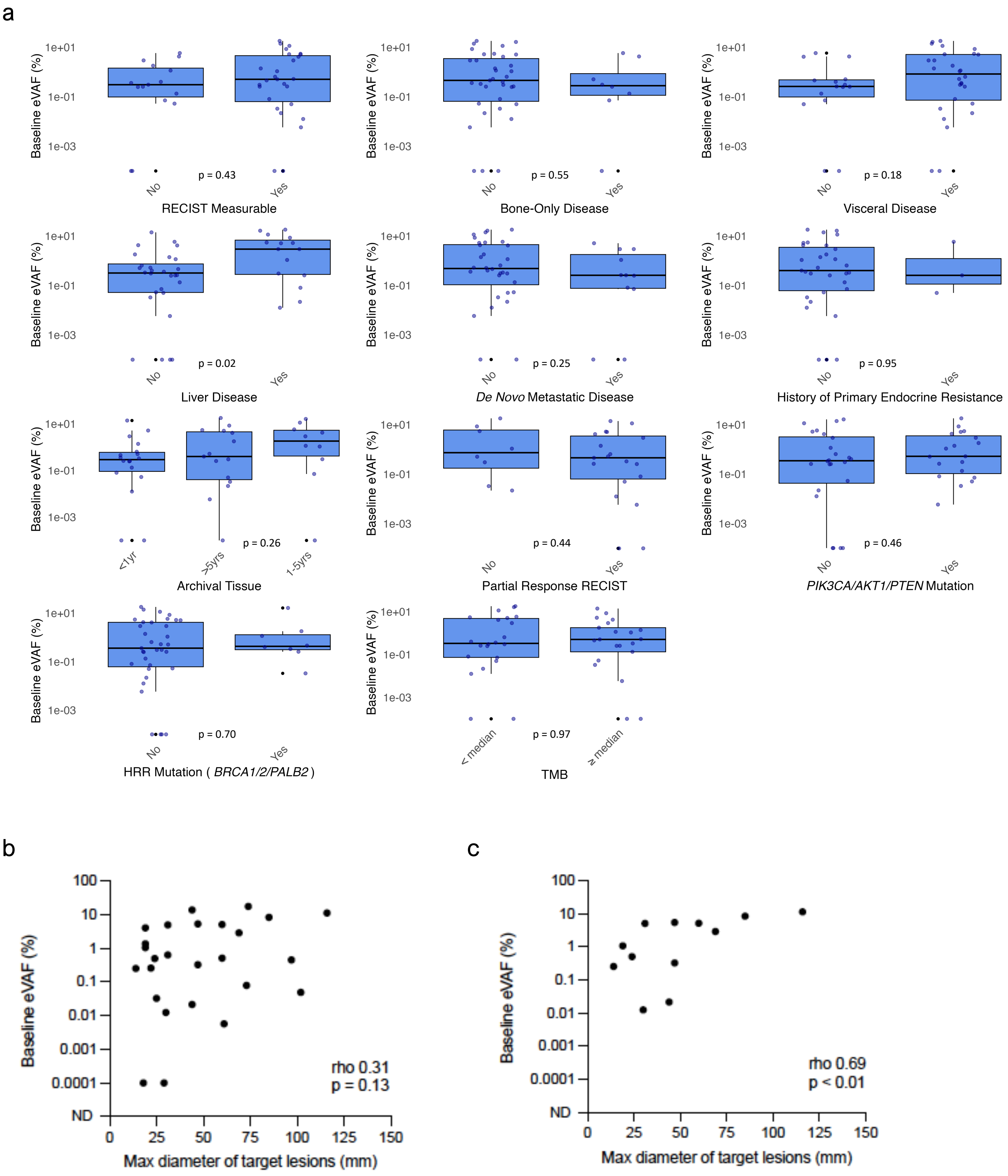

Supplementary Figure 2. (a) Clinicopathological and genomic associations with baseline eVAF, excluding failed samples. **(b)** Correlation of baseline eVAF with RECIST v1.1 target lesion measurements in the analytical cohort and **(c)** in patients with liver disease at baseline. **(d)** Time to treatment failure and **(e)** overall survival forest plots for univariable Cox models for baseline eVAF and key clinical variables including the presence of visceral disease, age at study entry, line of therapy, and ET pair on study. **(f)** Time to treatment failure and **(g)** overall survival forest plots for multivariable Cox models including baseline eVAF and the presence of visceral disease.

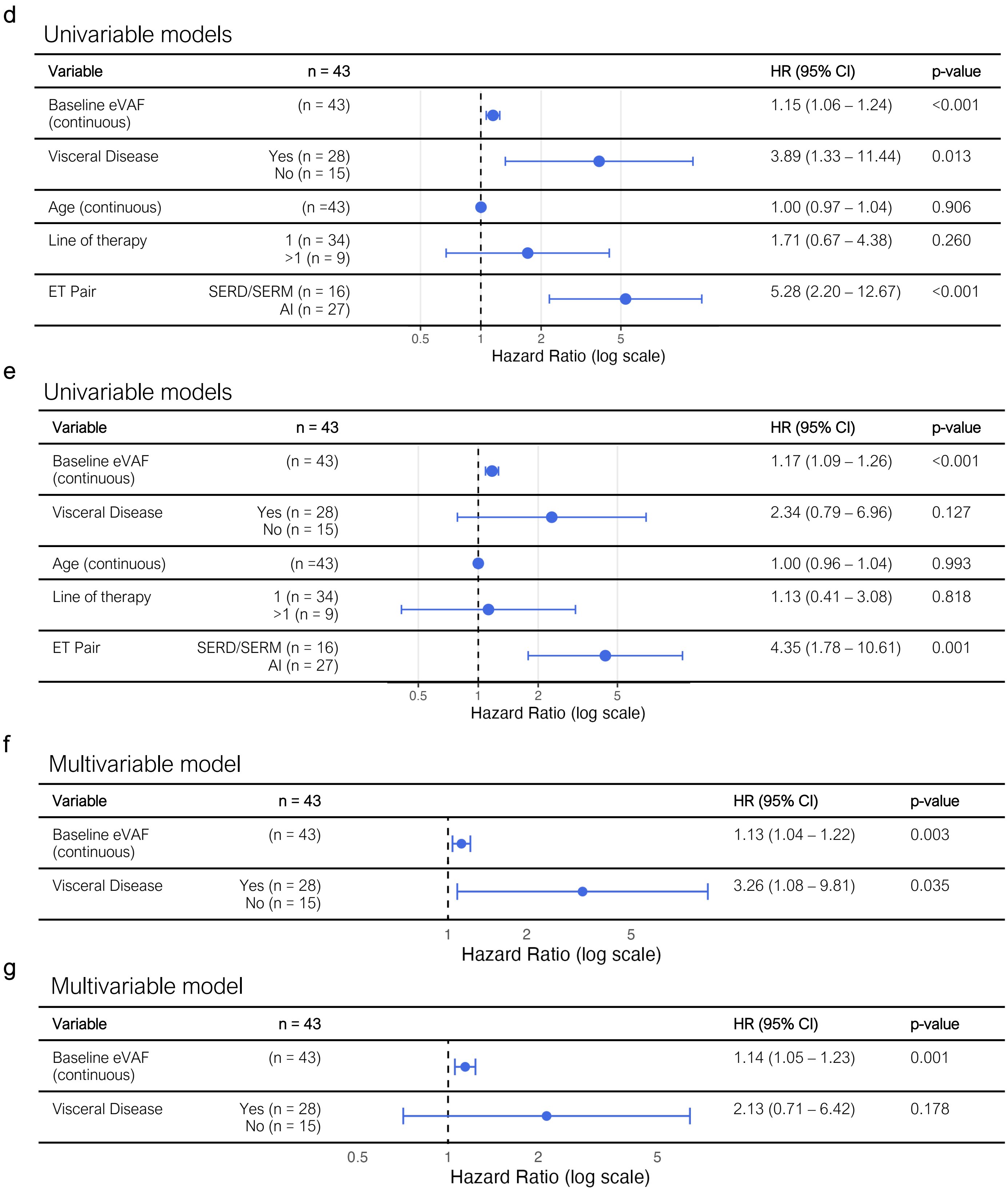

Supplementary Figure 3. (a) Individual plots of ctDNA kinetics until treatment failure or last follow-up. RECIST v1.1 response categories, time of radiological evaluations, ctDNA detection, and clinical outcomes are shown. Patients with primary resistance are indicated by a red arrow. **(b)** Individual plots of ctDNA kinetics until treatment failure or last follow-up with radiologist assessment shown. **(c)** Exploratory analysis of a patient with negative ctDNA samples using a refined calling pipeline to focus on 4 variants best supported by QC. **(d)** ctDNA kinetics during the first 90 days of treatment and RECIST v1.1 partial response. **(e)** Correlation of the changes in eVAF and RECIST v1.1 target lesion measurements at best response. **(f)** Case vignettes of patients with discordant changes in eVAF and RECIST v1.1 measurements at best response.

a

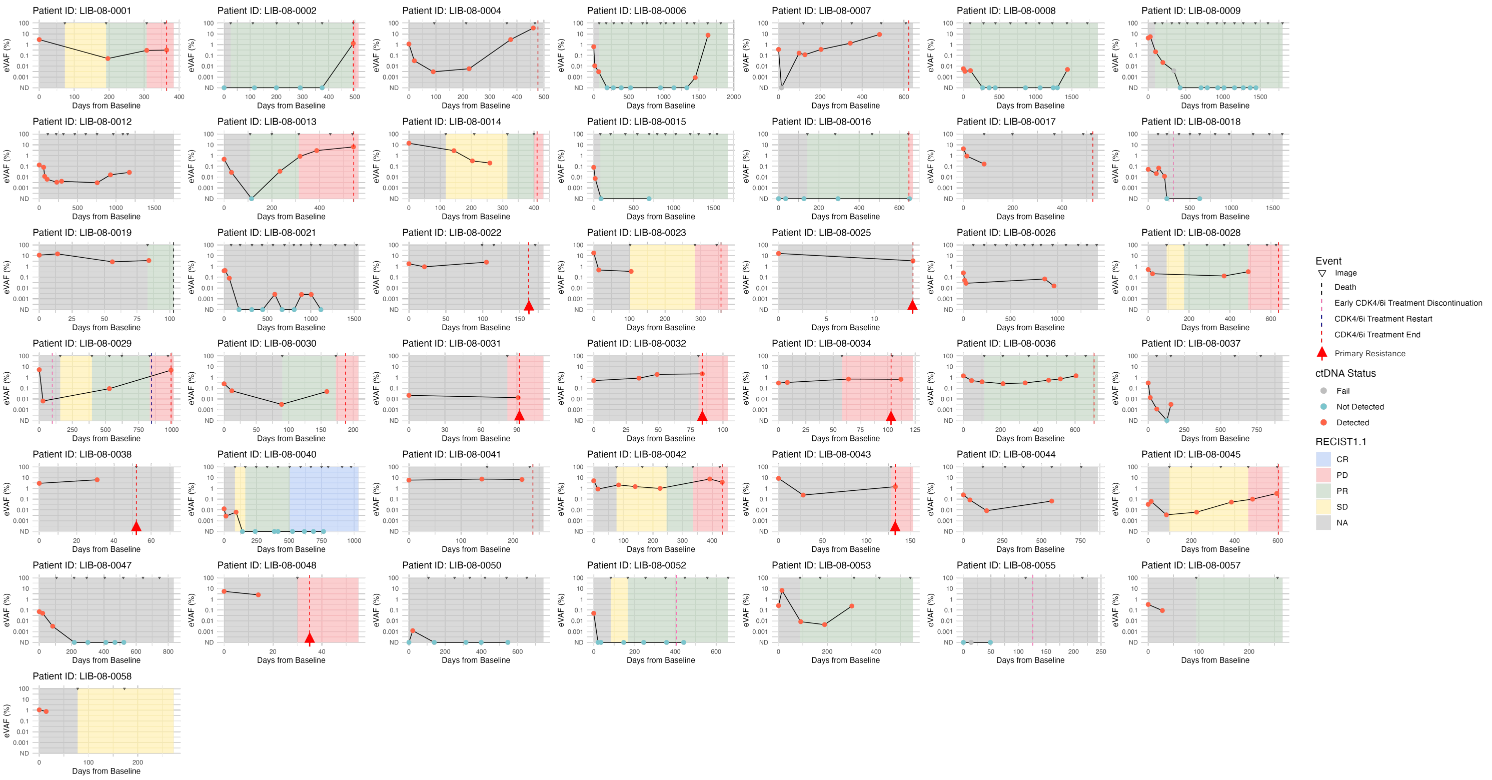

b

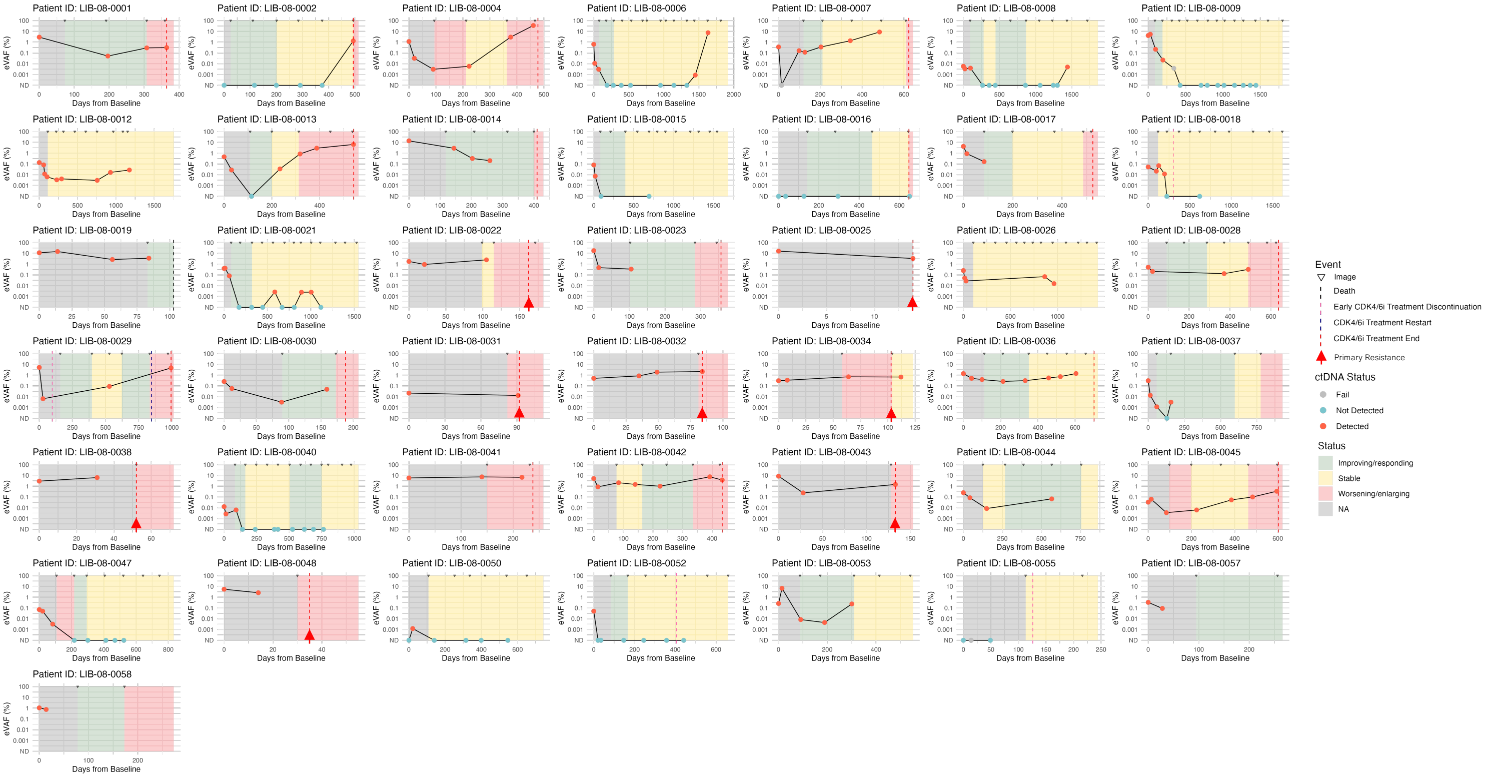

Supplementary Figure 3. (a) Individual plots of ctDNA kinetics until treatment failure or last follow-up. RECIST v1.1 response categories, time of radiological evaluations, ctDNA detection, and clinical outcomes are shown. Patients with primary resistance are indicated by a red arrow. **(b)** Individual plots of ctDNA kinetics until treatment failure or last follow-up with radiologist assessment shown. **(c)** Exploratory analysis of a patient with negative ctDNA samples using a refined calling pipeline to focus on 4 variants best supported by QC. **(d)** ctDNA kinetics during the first 90 days of treatment and RECIST v1.1 partial response. **(e)** Correlation of the changes in eVAF and RECIST v1.1 target lesion measurements at best response. **(f)** Case vignettes of patients with discordant changes in eVAF and RECIST v1.1 measurements at best response.

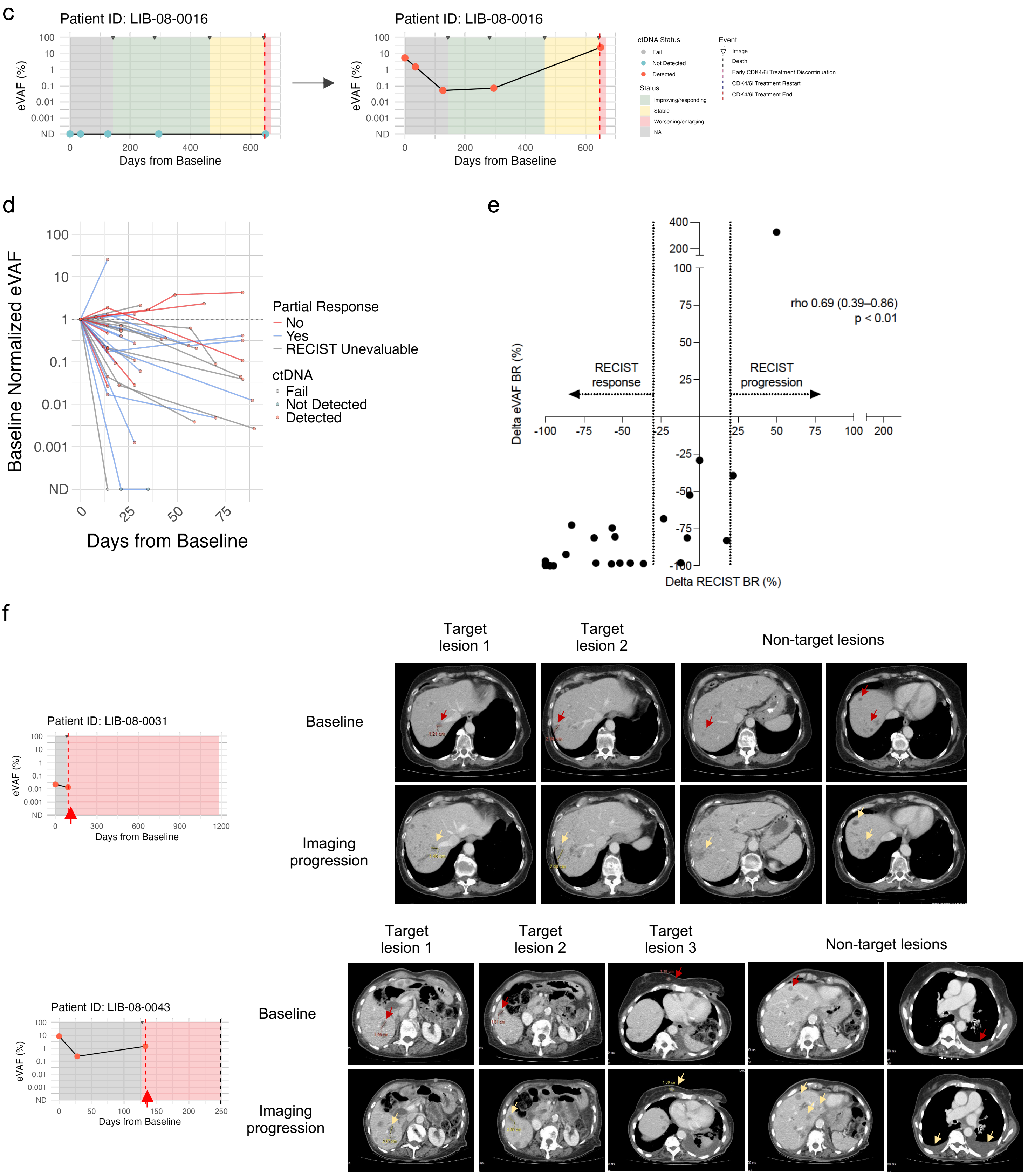

Supplementary Figure 4. (a) Time to treatment failure **(b)** and overall survival Cox models for ctDNA clearance, defined retrospectively based on the entire CDK4/6i treatment follow-up period, and key clinical variables including the presence of visceral disease, age at study entry, line of therapy and endocrine therapy pair on study. **(c)** Time to treatment failure **(d)** and overall survival according to ctDNA clearance at a 12-month landmark. **(e)** Clinicopathological associations of ctDNA clearance. **(f)** Time to treatment failure and **(g)** overall survival according to ctDNA clearance among RECIST v1.1 responders. **(h)** Time to treatment failure and **(i)** overall survival according to having more than 90% eVAF reduction from baseline ($eVAF_{<10\%}$), and their respective survival **(j, k)** at a 12-month landmark.

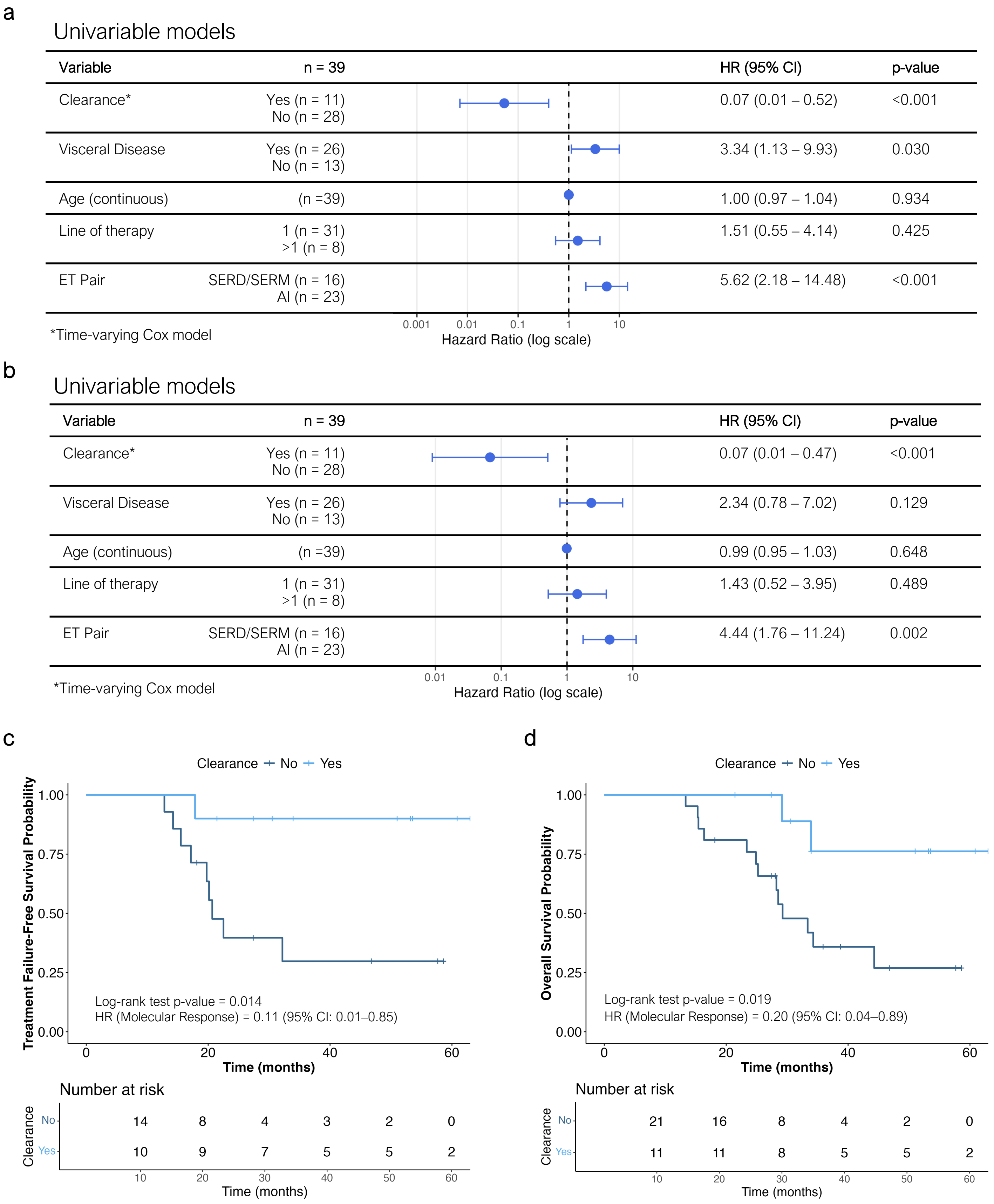

Supplementary Figure 4. **(a)** Time to treatment failure **(b)** and overall survival Cox models for ctDNA clearance, defined retrospectively based on the entire CDK4/6i treatment follow-up period, and key clinical variables including the presence of visceral disease, age at study entry, line of therapy and endocrine therapy pair on study. **(c)** Time to treatment failure **(d)** and overall survival according to ctDNA clearance at a 12-month landmark. **(e)** Clinicopathological associations of ctDNA clearance. **(f)** Time to treatment failure and **(g)** overall survival according to ctDNA clearance among RECIST v1.1 responders. **(h)** Time to treatment failure and **(i)** overall survival according to having more than 90% eVAF reduction from baseline ($eVAF_{<10\%}$), and their respective survival **(j, k)** at a 12-month landmark.

e

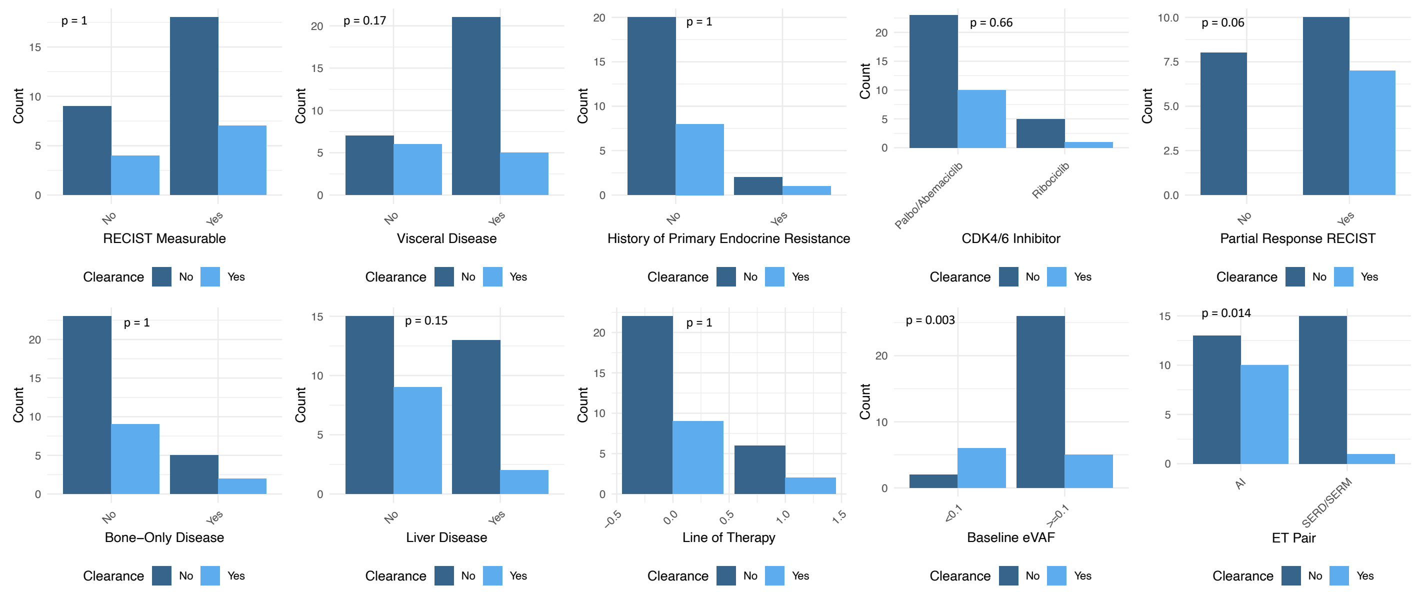

f

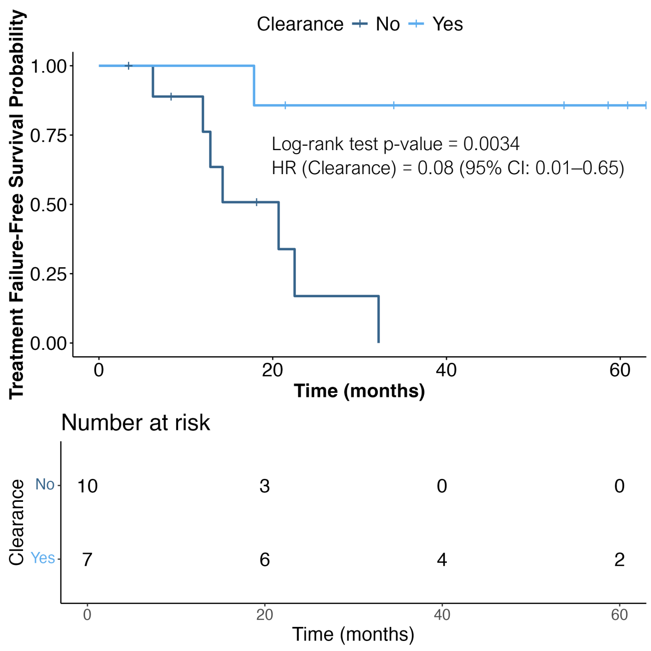

g

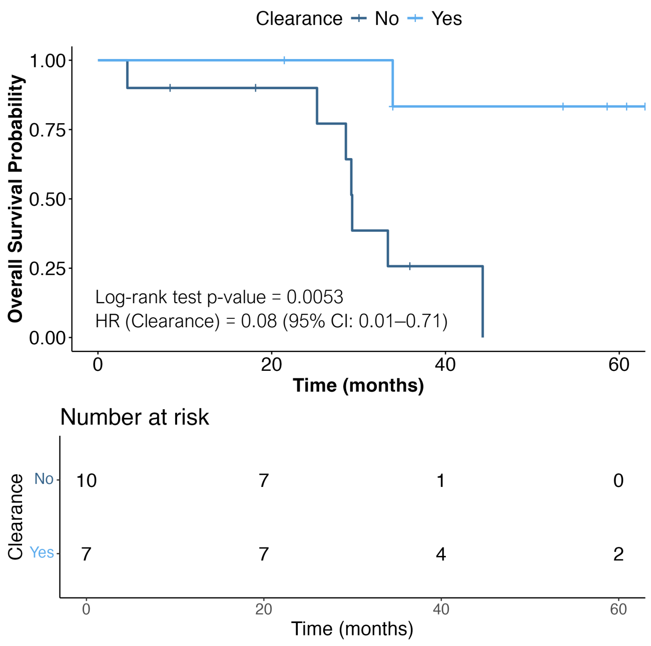

Supplementary Figure 4. (a) Time to treatment failure **(b)** and overall survival Cox models for ctDNA clearance, defined retrospectively based on the entire CDK4/6i treatment follow-up period, and key clinical variables including the presence of visceral disease, age at study entry, line of therapy and endocrine therapy pair on study. **(c)** Time to treatment failure **(d)** and overall survival according to ctDNA clearance at a 12-month landmark. **(e)** Clinicopathological associations of ctDNA clearance. **(f)** Time to treatment failure and **(g)** overall survival according to ctDNA clearance among RECIST v1.1 responders. **(h)** Time to treatment failure and **(i)** overall survival according to having more than 90% eVAF reduction from baseline ($eVAF_{<10\%}$), and their respective survival **(j, k)** at a 12-month landmark.

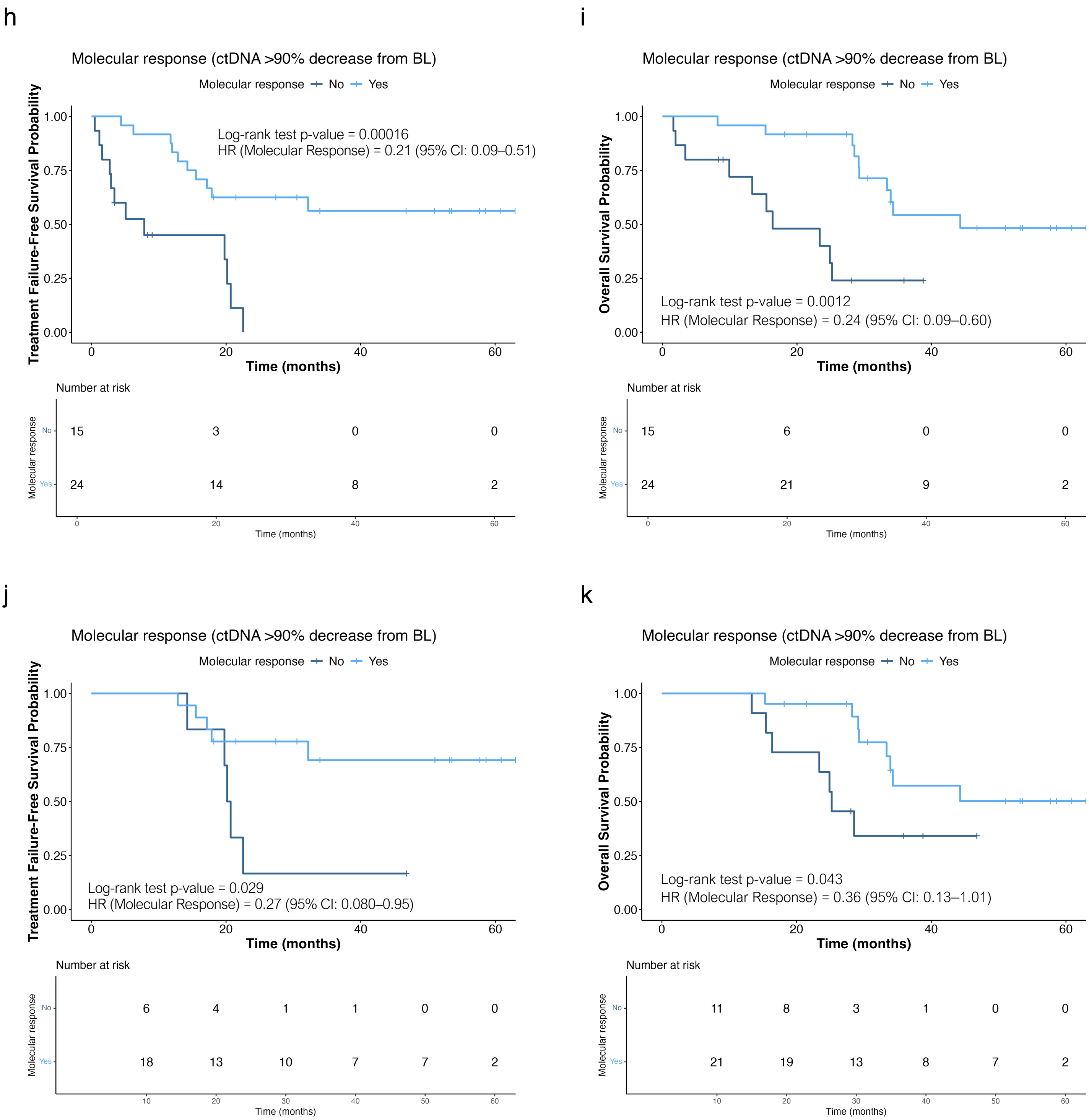

Supplement: Supplementary file 1 — Supplementary figures_ [file 41523_2025_783_MOESM1_ESM.pdf]
